# Gut microbial metabolites targeting JUN in renal cell carcinoma via IL-17 signaling pathway: network pharmacology approach

**DOI:** 10.1007/s11030-025-11188-5

**Published:** 2025-04-18

**Authors:** Stany Bala Kumar, Shatakshi Mishra, Anushka Das, Sagnik Nag, Rakesh Naidu

**Affiliations:** 1 School of Science, Monash University Malaysia, Jalan Lagoon Selatan, 47500 Bandar Sunway, Selangor Malaysia; 2 Jeffrey Cheah School of Medicine and Health Sciences, Monash University Malaysia, Jalan Lagoon Selatan, 47500 Bandar Sunway, Selangor Malaysia; 3 Department of Biomedical Sciences, School of Bio-Sciences & Technology (SBST), Vellore Institute of Technology (VIT), 632014 Vellore, Tamil Nadu India

**Keywords:** Renal cell carcinoma, JUN/AP-1, IL-17 pathway, Gut microbial metabolites, Icaritin, Molecular docking

## Abstract

The gut microbiome plays a crucial role in renal diseases, influencing conditions such as renal cell carcinoma (RCC), acute kidney injuries, and diabetic nephropathy. Recent studies highlight the association between gut microbial metabolites (GMM) and RCC progression. This study employs a computational network pharmacology framework to explore the mechanistic action of gut microbiota-derived metabolites against RCC. GMM were selected from the gutMgene database and analyzed for common targets using DisGeNET, Gene Card, and OMIM. Downstream analysis included gene ontology, KEGG pathway enrichment, metabolite-target-pathway-disease network construction, and protein–protein interaction analysis. Further, key metabolites were evaluated for drug-likeness, ADMET properties, and molecular docking, followed by molecular dynamics simulations (MDS) to assess complex stability. The JUN/AP-1 gene emerged as the prime target, exhibiting the highest binding affinity with Icaritin (− 5.9 kcal/mol), followed by Quercetin and Luteolin. MDS confirmed the stable binding of Icaritin to the active site throughout the simulation. These GMM may influence anticancer activity through distinct regulatory pathways involving the JUN/AP-1 gene, either by inhibiting or modulating its function. These insights establish a basis for further in vitro and in vivo investigations, supporting the development of microbiome-based therapeutic approaches.

**Graphical abstract:**

The graphical abstract illustrates a computational network pharmacology and computer-aided analysis approach to investigating gut microbiota-derived metabolites in renal cell carcinoma (RCC). It highlights core target genes, gene ontology, KEGG pathways, and network analyses. The right section depicts gut microbiota, specifically *Bacterium sp. MRG-PMF-1* produces Icaritin, which inhibits the JUN gene, potentially suppressing RCC progression. Molecular docking and molecular dynamics simulations confirm stable binding interactions, supporting Icaritin’s therapeutic potential.

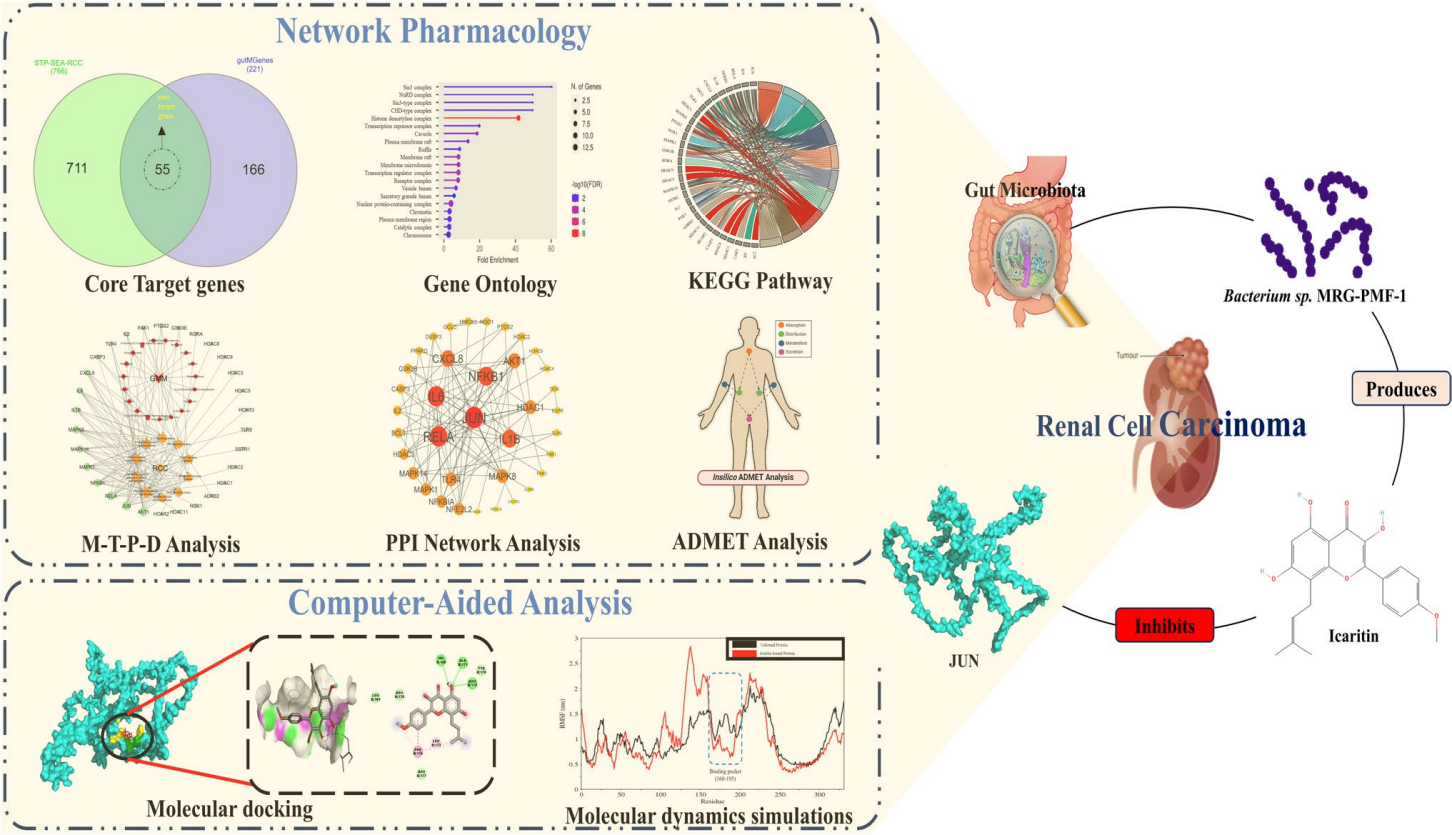

**Supplementary Information:**

The online version contains supplementary material available at 10.1007/s11030-025-11188-5.

## Introduction

Renal cell carcinoma (RCC) remains the 14th most common cancer worldwide, with 434,840 new cases reported in 2022 [1]. The highest incidence rates of kidney cancer were seen in China, the United States, and Russia [2, 3]. Recent studies have also identified 50 new genomic regions associated with the risk of RCC, improving understanding of the disease’s molecular mechanisms and offering insights for early detection and potential new treatments [4]. Effective management and intervention strategies are essential for improving outcomes and reducing mortality in RCC. Early detection and timely treatment are crucial in slowing disease progression, underscoring the need for comprehensive, targeted therapies for RCC. Current treatments for RCC often include targeted therapies like tyrosine kinase inhibitors (e.g., Sunitinib, Pazopanib) [5], mTOR inhibitors (e.g., Everolimus) [6], VEGF inhibitors (e.g., Bevacizumab) [7], and immunotherapies (e.g., Nivolumab, Pembrolizumab) [8]. These drugs, while effective, are associated with significant side effects such as fatigue, hypertension, diarrhea, skin rash, and more severe complications like pneumonitis and liver inflammation [9]. The high incidence of severe side effects emphasizes the need for alternative therapies. Natural products and microbial compounds offer safer, sustainable treatments with unique actions, potentially enhancing efficacy and reducing drug resistance in oncology.

The gut microbiota supports health by producing vitamins, regulating cholesterol, and generating short-chain fatty acids (SCFAs) for gene regulation. Dietary fibers sustain microbial diversity, supporting phyla like Bacteroidetes and Firmicutes, which aid in pathogen defense through antimicrobial secretion, nutrient competition, and site occupation [10]. Dysbiosis is linked to diseases such as Crohn’s disease [11], type 1 diabetes [12], and obesity [13]. Microbial metabolites, like indole derivatives and bile acids, influence bacterial behavior and growth, with some (e.g., bacteriocins) showing antibiotic potential [14]. SCFAs and bile acids improve energy metabolism and appetite regulation, while dysregulation of metabolites links to obesity [15], type 2 diabetes [16], and cardiovascular disorders [17]. *Fusobacterium* and *Porphyromonas* were found to be associated with an increased risk of colorectal cancer (CRC) in studies by Ahn et al. and Wei et al., with the latter demonstrating *Fusobacterium* nucleatum’s ability to increase tumor multiplicity and induce pro-inflammatory responses [18, 19]. Other bacteria like *Bacteroides fragilis*, *Streptococcus gallolyticus*, and *Prevotella* have also been linked to increased CRC risk [19, 20]. Various studies have elucidated the complex relationship between gut microbiota and the development of cancer, particularly RCC [21], CRC [22], and other types like liver [23] and breast cancer [24]. According to a study conducted by Derosa et al. gut microbiota composition, altered by antibiotics and tyrosine kinase inhibitors, affects immune checkpoint blockade (ICB) therapy outcomes in RCC patients. Beneficial bacteria like *Akkermansia muciniphila* may serve as biomarkers for predicting ICB success [25]. In patients with metastatic RCC receiving checkpoint inhibitor therapy, having a greater variety of gut microbes, especially more of a specific bacterium called *Akkermansia muciniphila*, is linked to better treatment results. [26]. In patients with metastatic RCC, adding CBM588, a bacterial supplement that promotes the growth of *Bifidobacterium*, to nivolumab and ipilimumab treatment did not noticeably increase gut bacteria diversity or the amount of *Bifidobacterium*. However, the group receiving CBM588 showed much longer progression-free survival (12.7 months compared to 2.5 months) and a slightly better response rate, though the response rate difference wasn’t statistically significant [27]. Therefore, the gut microbiome plays a crucial role in the effectiveness of checkpoint inhibitor therapies for cancer, with potential benefits from enhancing microbial diversity or using probiotics. Further research is needed to confirm and optimize these therapeutic strategies.

**Fig. 1:**
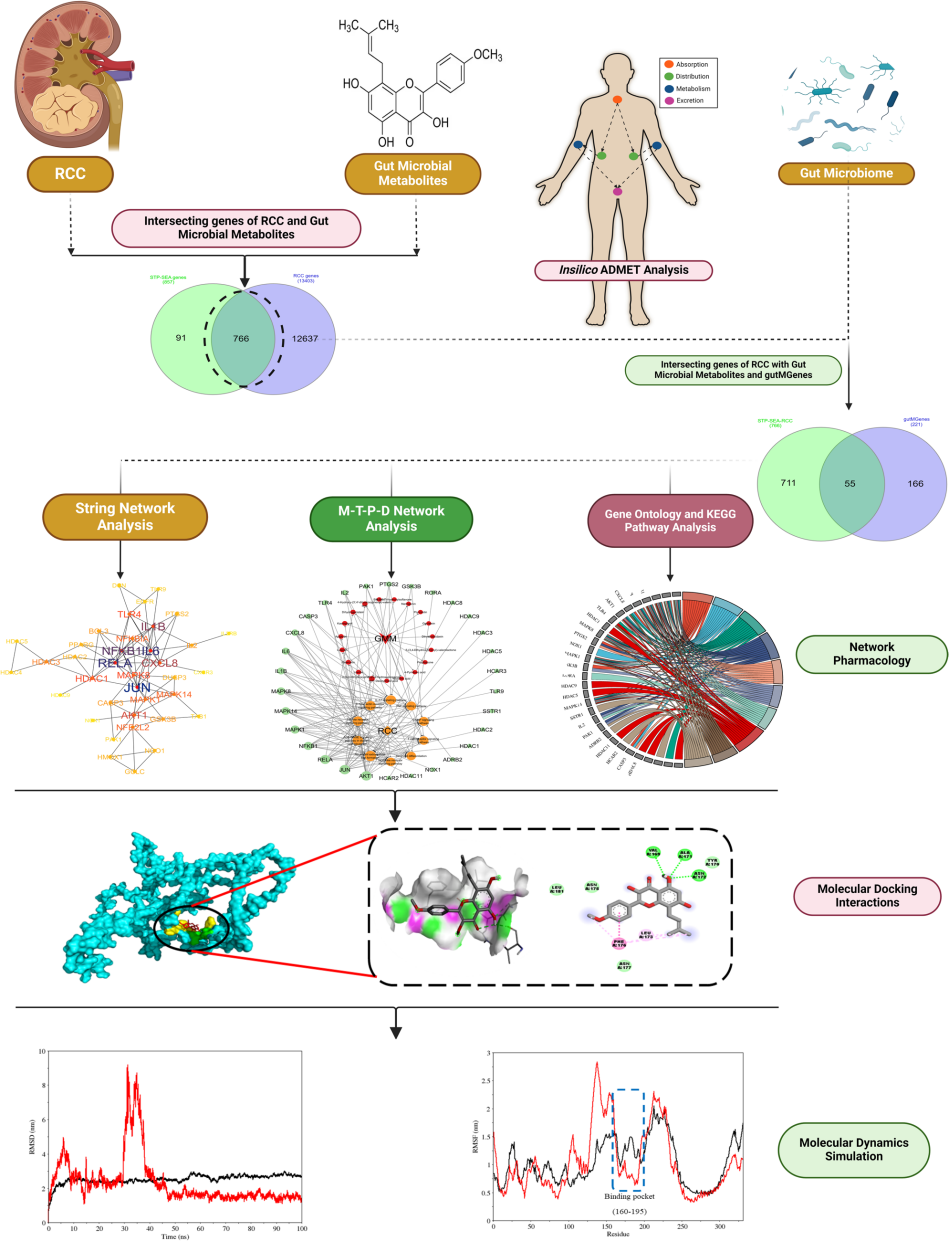
Research workflow for identifying therapeutic targets in RCC via GMM interactions: 766 common genes between RCC and GMM were identified, from which 55 core target genes were selected for GO and KEGG enrichment analysis. The top 10 KEGG pathways were used for MTPD network analysis, followed by PPI network construction. ADME/T screening was performed on 184 GMM, with molecular docking and molecular dynamics simulations used to evaluate interactions between the prime target gene and the highest binding energy GMM

The present study investigates the role of gut microbial metabolites (GMM) in renal cell carcinoma (RCC) by targeting RCC-related genes using an *in-silico* approach with various bioinformatics tools Table S1 (Supplementary File). The workflow illustrated in Fig. 1 outlines each research phase, including identifying common targets between RCC and GMM genes via network pharmacology, gene ontology, KEGG pathway enrichment, M-T-P-D network analysis, and protein–protein interactions (PPI). Findings from PPI analysis undergo molecular docking and are assessed for drug-likeness and ADME/T properties, with binding affinities validated by molecular dynamics simulation (MDS). This outcome helps establish a link between the prime target gene and the key RCC pathway. Nevertheless, this pursuit remains a multifaceted challenge that demands extensive and rigorous in vitro and in vivo research.

## Methodology

### Retrieval of GMM and gutMgene targets

GMM and gutMgene targets associated with *Homo sapiens* are sourced from the gutMGene database (http://bio-annotation.cn/gutmgene/browse.dhtml) (accessed on 18 March 2024) [28]. The genes associated with GMM are extracted using the SEA (https://sea.bkslab.org/) (similarity ensemble approach) (accessed on 18 March 2024) search server [29], and Swiss Target Prediction (STP) databases (http://www.swisstargetprediction.ch/) (accessed on 18 March 2024) [30]. The common genes between SEA and STP were identified using the InteractiVenn tool (https://www.interactivenn.net/) [31].

### Sourcing and screening of genes involved in RCC

To identify key genes involved in RCC and understand its molecular mechanisms, a comprehensive list will be compiled of RCC-associated genes using multiple databases. This will be formed as the basis for further analyses, including pathway enrichment and gene network construction, to focus on the most relevant therapeutic targets. The genes involved in RCC are obtained from databases like DisGeNET (https://www.disgenet.org/search) (accessed on 18 March 2024) [32], GeneCards (https://www.genecards.org/) (accessed on 18 March 2024) [33], and OMIM (https://www.omim.org/) (Online Mendelian Inheritance in Man) (accessed on 18 March 2024) [34] by using “renal cell carcinoma” as the keyword. The genes associated with RCC, obtained from relevant databases, and the common genes from STP-SEA analysis were considered to further identify shared genes between the two entities. These common genes were then compared with gutMgene target datasets to identify core target genes.

### GO and enrichment analysis of the selected genes

The key RCC-associated genes will undergo analysis using Gene Ontology (GO) and enrichment analyses to assess their biological relevance. This process will identify the cellular components, biological processes, and molecular functions associated with these genes [35], offering insights into their potential role in RCC and informing subsequent network analyses. The core target genes are subjected to enrichment analysis to understand the associated metabolic pathways using ShinyGO 0.80 (http://bioinformatics.sdstate.edu/go/) [36]. The genes are examined for their involvement in the cellular components, biological processes, and molecular functions maintaining a false discovery rate (FDR) cutoff of 0.05, ensuring statistical significance of the findings. Redundancy in the data was eliminated to focus on the most relevant pathways [37], and the analysis was limited to the species “*Homo sapiens*”. A lollipop plot was generated to illustrate the involvement of the selected genes in various cellular components, biological processes, and molecular functions, as derived from the data.

### M-T-P-D network analysis of key KEGG-enriched pathway genes

To explore the relationships among metabolites, core target genes, pathways, and diseases by constructing a Metabolite-Target-Pathway-Disease (M-T-P-D) network. Integrating metabolomic and genetic data will aid in identifying potential therapeutic targets and pathways for RCC treatment. Cytoscape v3.10.2 [38] was used to construct a comprehensive M-T-P-D network model. The model was developed by integrating the top 10 enriched pathways identified from the KEGG pathway analysis [37, 39]. These pathways were utilized to establish relationships among the metabolites, targets, pathways, and diseases, thereby creating a complete regulatory network. The internal ranking within this network was established based on the degree value of the components.

### PPI network analysis of the core target genes

PPI network analysis is used to identify hub genes and their encoded proteins obtained from core target gene set which are critical for network structure and function [40]. This step bridges gene screening, pathway analysis, and molecular docking, to ensure only the most relevant genes are prioritized. The core target genes screened from the above steps are subjected to the STRING database v12.0 (https://string-db.org/) to attain the highly connected genes within a biological network that play key roles in regulating the network’s stability and function [41]. A confidence interval of 0.900 was maintained, to ensure reliable interactions among genes. The resulting data were visualized using the Cytoscape v3.10.2, where genes were ranked based on their degree score highlighting those with the highest connectivity within the network. The genes were further analyzed using cytoHubba for the identification of the top 10 hub genes using key centrality measures like MNC, degree, and closeness [42]. Hub genes are the highly connected genes within a network, identified through topological measures [43]. The analysis included a detailed evaluation of the shortest paths and highest score of each gene involved in the network, to provide deeper insights into the selection of prime target gene that may play pivotal roles in the biological processes under study.

### ADME/T screening of GMM

ADME/T (absorption, distribution, metabolism, excretion, and toxicity) screening is used to evaluate the pharmacokinetic properties of metabolites to ensure their suitability for drug development [44]. This step filters out unsuitable compounds, allowing only drug-like metabolites to proceed to molecular docking with prime target gene. The GMM sourced from gutMGene are examined for their, ADME/T using SwissADME (http://www.swissadme.ch/index.php) (accessed on 18 March 2024) [45], molsoft (https://molsoft.com/mprop/) (accessed on 18 March 2024), and ADMETlab 2.0 (https://admetmesh.scbdd.com/) (accessed on 18 March 2024) [46]. The initial screening of metabolites was conducted based on Lipinski’s Rule of Five, which is used to assess drug-likeness by evaluating key parameters: molecular weight (< 500 g/mol), lipophilicity/logP (< 5), the number of hydrogen bond acceptors (< 10), hydrogen bond donors (< 5), and rotatable bonds (< 10) [47]. Following this, the metabolites were further screened for bioavailability, focusing on a bioavailability score of ≥ 0.55 and a topological polar surface area (TPSA) value of ≤ 140.

### Molecular docking of the prime target gene and metabolites

Molecular docking is performed to evaluate the binding affinity of selected metabolites to the identified prime target gene. This step validates the interaction between drug-like metabolites and prime target gene, offering insights into their therapeutic potential for RCC. The structure of the prime target gene was retrieved from the Alpha-Fold and docked with the metabolites qualifying the ADME/T analysis. The active sites are found using the CASTp server v3.0 (http://sts.bioe.uic.edu/castp/index.html?2was) (accessed on 18 March 2024) [48]. The docking is executed at AutoDockTools 1.5.7 [49], with the grid box parameters set appropriately for the binding site of the target protein. The 2D and 3D interactions are visualized using Discovery Studio and PyMOL respectively [50].

### Molecular dynamics simulations

Following molecular docking, MDS is employed to assess the stability and behavior of metabolites in a dynamic biological environment. This step is evaluated for the flexibility and interactions of the metabolite within the biological system [51], confirming its potential therapeutic efficacy for RCC and validating the stability of the metabolite-gene complex. MDS was conducted using Gromacs version 2023.1 to assess the stability of the metabolite during the simulation. The Charmm36 force field was utilized, and the metabolite’s topology was generated via the CGenFF webserver. Solvation was achieved with the SPC (Simple Point Charge) water model within a dodecahedron simulation box, including explicit water molecules. 0.15 M NaCl (29 Na + ions, 27 Cl − ions) was incorporated to ensure charge neutrality [52, 53]. The system was subjected to an energy minimization process with the steepest descent algorithm for 50,000 steps, followed by equilibration under NVT (number of molecules, volume, temperature) and NPT (number of molecules, pressure, temperature) ensembles at 300 K and 1 bar pressure. MDS was performed for 5000 frames per simulation, each lasting 100 ns, using the leap-frog MD integrator. The simulation trajectory was calculated and analyzed using root mean square deviation (RMSD), root mean square fluctuations (RMSF), Radius of Gyration (Rg), and Solvent accessible surface area (SASA).

## Results and discussion

### Screening the target genes of metabolites

**Fig. 2:**
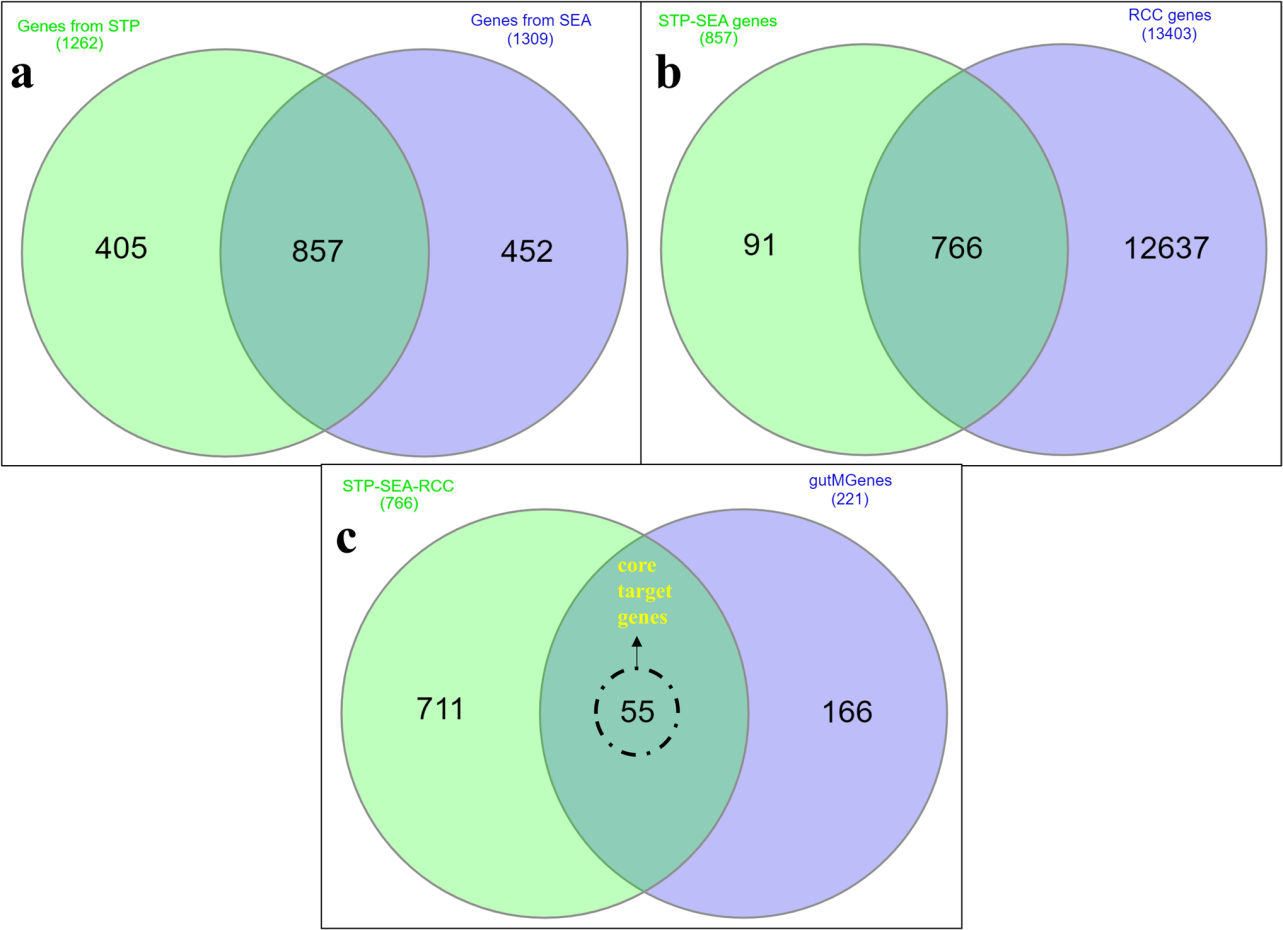
Screening of the target genes for network pharmacology. **A** Common metabolite genes obtained from STP and SEA servers. **B** Common genes between STP-SEA overlap and RCC genes. **C** Analysis of the core target genes between STP-SEA-RCC overlap and gutMgene targets

The analysis probed into an exploration of 184 metabolites, from distinct sources. Specifically, 1262 genes were derived from the STP, and 1309 genes were identified through SEA. The genes selected from the SEA and STP datasets are shown in Fig. 2A as a Venn diagram, where 857 genes were methodically selected from this initial pool, and non-repetitive genes from both datasets were eliminated.

### Screening of the target genes for RCC

The RCC genes were curated using DisGeNET, Gene Card, and OMIM, yielding 873, 13225, and 186 genes, respectively, which led to a total of 13403 screened genes. To streamline the analysis, detailed scrutiny identified STP-SEA-RCC (766) genes that were common among the STP-SEA (857) genes associated with GMM and the extensive set of 13403 genes linked to RCC as shown in Fig. 2B**.** The 766 common genes were examined against the gutMgene targets (221) associated with the human gut. This resulted in the identification of 55 core target genes that emerged as the focal point of interest, as illustrated in Fig. 2C. These core target genes form a targeted subset with potential implications for further understanding the complex association between GMM and genes relevant to RCC.

### GO and enrichment analysis of the core target genes

**Fig. 3:**
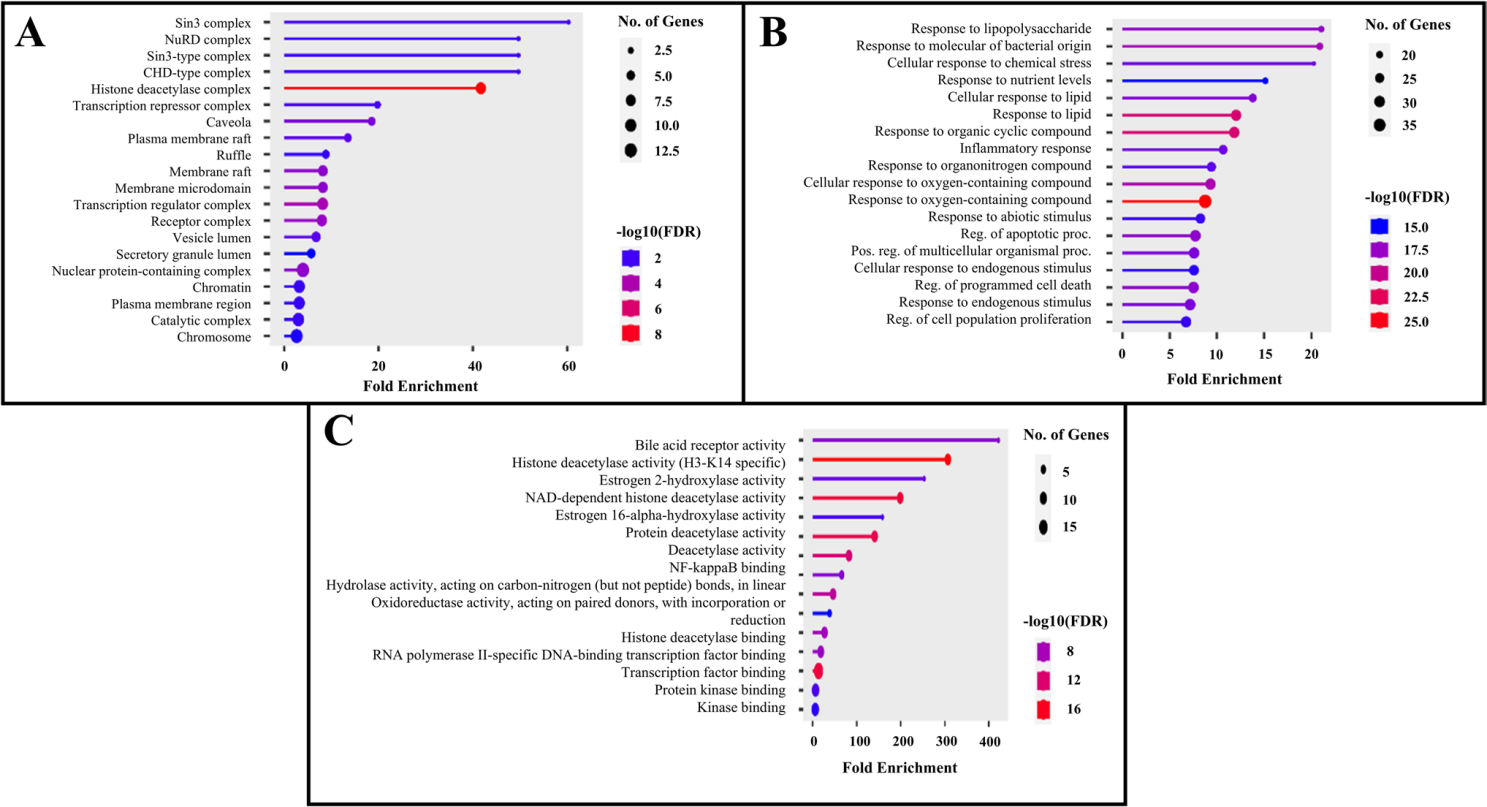
Pictorial representation of the GO analysis representing the functional classification of genes based on their associated biological roles and activities. **A** Cellular components **B** Biological process **C** Molecular function

The GO analysis of the 55 core target genes is presented in Fig. 3A, B and C, covering cellular components, biological processes, and molecular functions. Among cellular components, the Sin3 complex shows the highest fold enrichment, followed by the NuRD and Sin3-type complexes. Table S2 (Supplementary Table) highlights that, in terms of biological processes, most genes are associated with the response to lipopolysaccharide (LPS), responses to molecules of bacterial origin, and cellular responses to chemical stress. For molecular functions, the genes are predominantly involved in bile acid receptor activity, histone deacetylase activity (H3-K14 specific), and estrogen 2-hydroxylase activity. The Sin3 complex, a key cellular component, regulates the cell cycle, proliferation, and differentiation. Its dysregulation has been associated with cancer development [54]. Brito et al. reported that the downregulation of Sin3B plays a role in the clearance of RCC [55]. Additionally, activation by LPS triggers HMGB1 expression, a protein linked to inflammation and kidney cancer development [56]. Furthermore, bile acids can dysregulate the farnesoid X receptor (FXR) and G protein-coupled bile acid receptor 1 (TGR5), promoting uncontrolled cell proliferation and tumor growth [57].

**Fig. 4:**
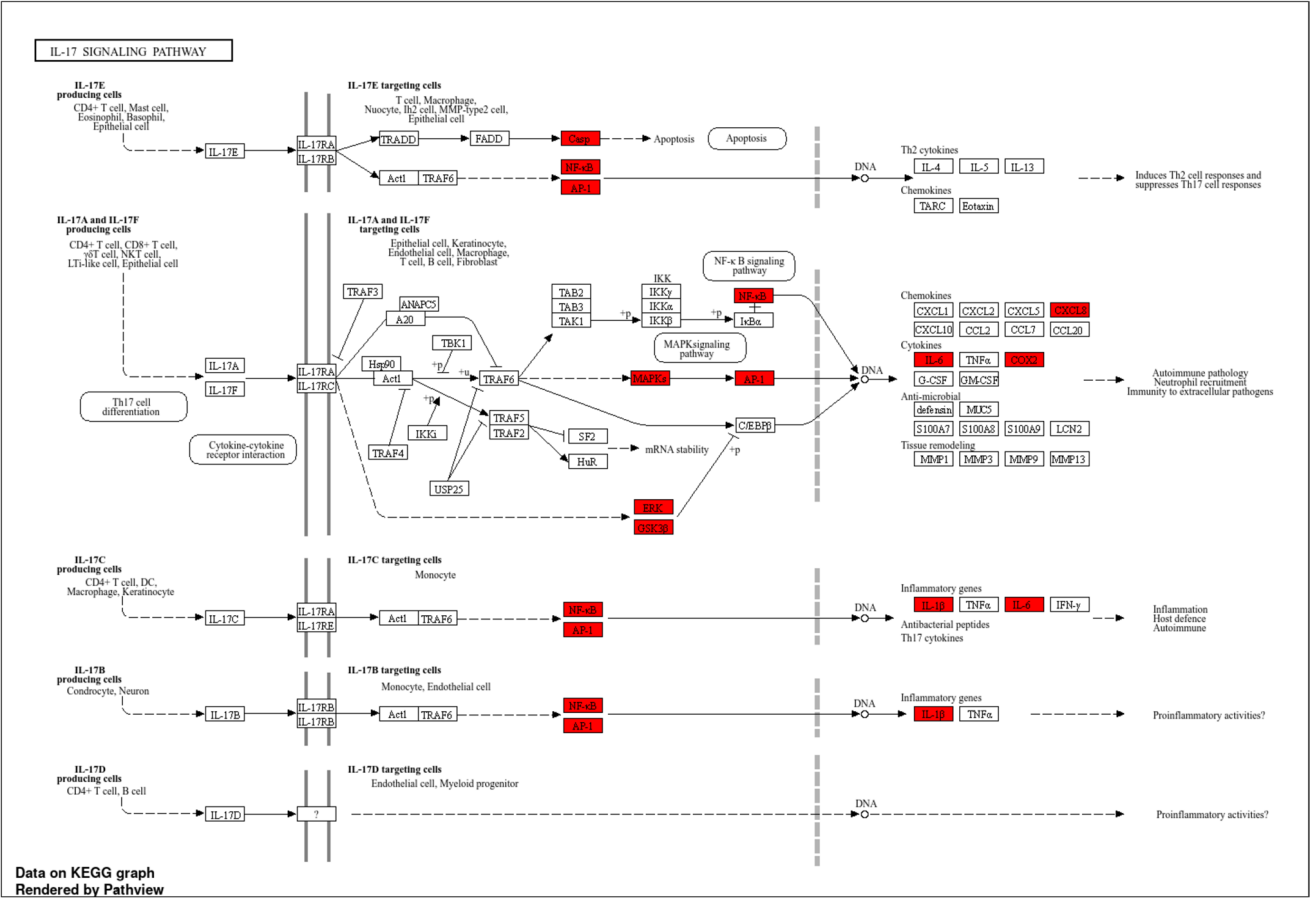
IL-17 signaling pathway illustrating the involvement of core target genes in the pathway’s regulatory and signaling processes

**Fig. 5:**
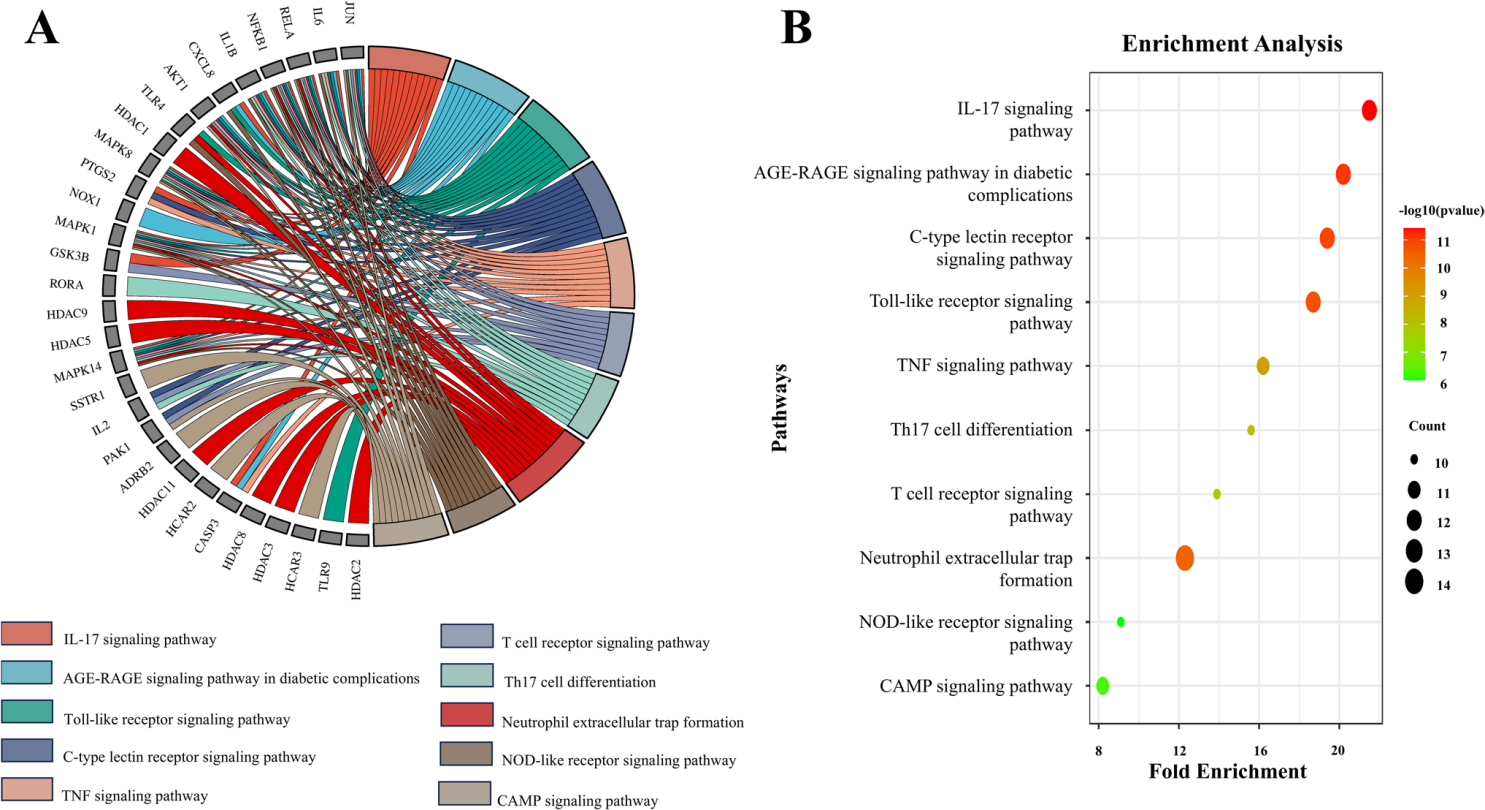
Enrichment analysis of the top 10 KEGG pathways associated with RCC. **A** Chord plot showing the relationship between RCC-related genes and enriched KEGG pathways **B** Bubble plot illustrating the significance of KEGG pathway with bubble size reflecting gene count

A pathway enrichment analysis was conducted to explore the functional implications of the 55 core target genes, with the top ten pathways ranked in Table S3 (Supplementary file), highlighting their relevance to RCC. The IL-17 signaling pathway stands out, with a significant fold enrichment value of 54.47 and namely, PTGS2, GSK3B, MAPK1, MAPK8, NFKB1, MAPK14, IL1B, IL6, CASP3, CXCL8, RELA, JUN/AP-1 involved (Fig. 4). To aid interpretation, a chord plot (Fig. 5A) illustrates gene interactions within this pathway, while a bubble plot (Fig. 5B) visually organizes pathway hierarchies, where bubble size correlates with significance. The IL-17 signaling pathway, identified as a critical area for further research, is driven by IL-17, a pro-inflammatory cytokine produced by T cells, which acts in various tissues, including the kidneys, playing a prominent role in inflammation and cancer development [58]. IL-17 recruits immune cells such as neutrophils and macrophages within tumors, influencing tumor growth and immune responses [59]. IL-17, predominantly secreted by Th17 cells, functions as a pivotal role in driving cancer progression by promoting tumor cell proliferation, enhancing angiogenesis, and aiding metastasis, thereby accelerating the overall advancement of malignant growth. [60]. Understanding the role of IL-17 in RCC could offer opportunities for targeted therapies to inhibit this pathway, potentially slowing or preventing disease progression.

### M-T-P-D network analysis of top 10 KEGG-enriched pathway genes

**Fig. 6:**
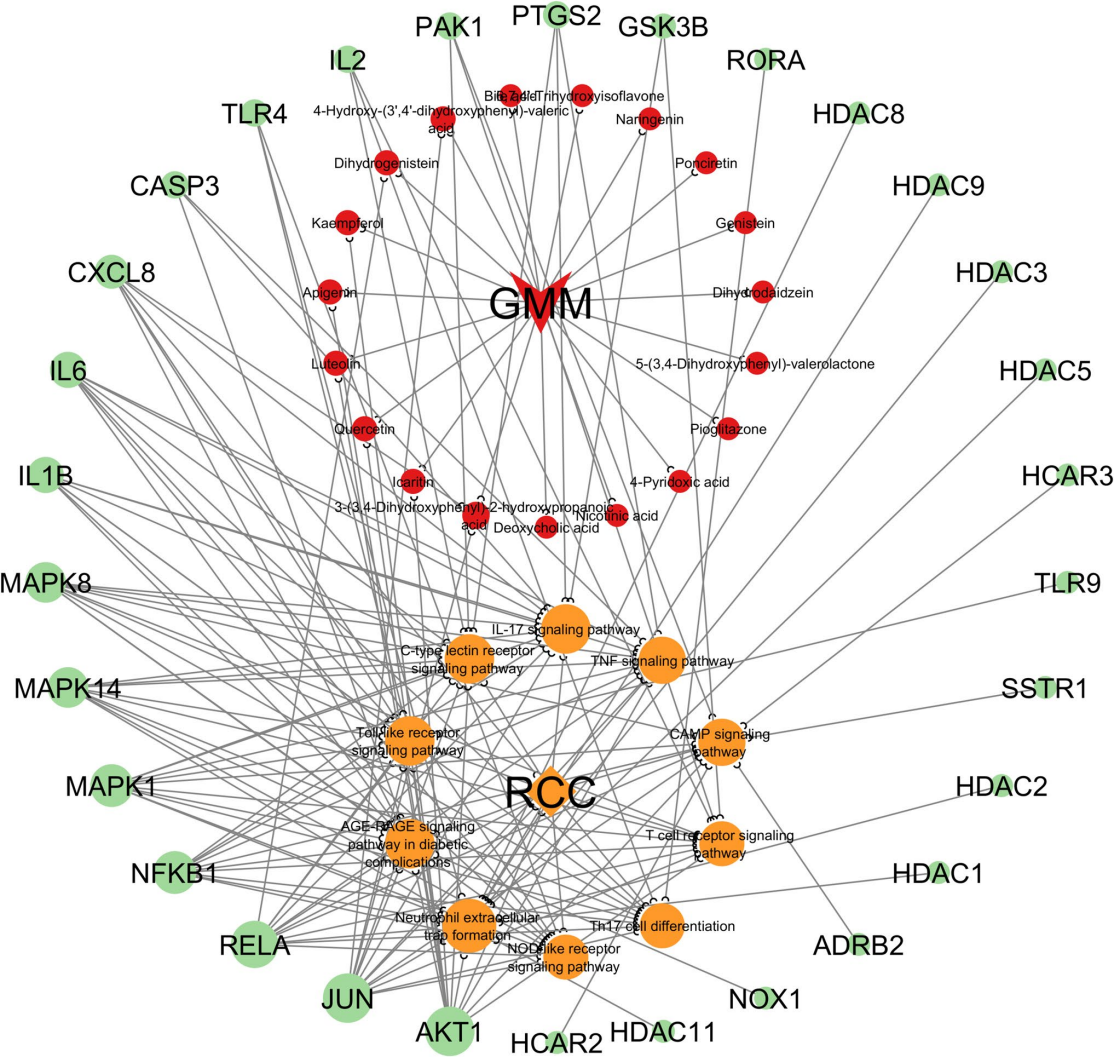
Metabolite-target-pathway-disease network illustrating the potential mechanism of GMM against RCC, highlighting key metabolites, targets, and pathways involved

The M-T-P-D network of GMM for treating RCC underscores the complex interactions between GMM and RCC. The network was constructed using the top 10 KEGG pathway genes associated with the GMM to explore the relationships between these genes, the corresponding pathways, and the metabolites linked to each gene. The network consists of 61 nodes, including 10 core pathways, 30 target nodes, 19 compound nodes, 1 GMM node, and 1 RCC node, interconnected by 156 edges as illustrated in Fig. 6. In the network, target nodes (genes) appear as green circles, while metabolites and the GMM are depicted as red circles and a red prism, respectively. The top 10 pathways are shown as orange circles, and an orange diamond represents RCC. Among all the pathways, genes, and compounds analyzed, the IL-17 signaling pathway, neutrophil extracellular trap formation, C-type lectin receptor signaling pathway, Toll-like receptor signaling pathway, and AGE-RAGE signaling pathway in diabetic complications, along with the genes JUN and AKT1, exhibited the highest degree score of 13. Similarly, compounds such as Icaritin, 3-(3,4-Dihydroxyphenyl)-2-hydroxypropanoic acid, 4-Hydroxy-(3′,4′-dihydroxyphenyl)-valeric acid, Dihydrogenistein, Kaempferol, Apigenin, Luteolin, and Quercetin also showed the highest degree score of 2. The detailed results are tabulated in Table S4 (Supplementary file). The M-T-P-D network analysis found that Icaritin, 3-(3,4-Dihydroxyphenyl)-2-hydroxypropanoic acid, 4-Hydroxy-(3’,4’-dihydroxyphenyl)-valeric acid, Dihydrogenistein, Kaempferol, Apigenin, Luteolin, and Quercetin were discovered as the most potential components of GMM against RCC. Li et al. found that Icaritin, a prenyl flavonoid derivative, exhibits potent antitumor activity in RCC by targeting the JAK2/STAT3 signaling axis. STAT3, constitutively activated in RCC, promotes tumorigenesis by enhancing proliferation, angiogenesis, and immune evasion. Icaritin inhibits both constitutive and IL-6-induced STAT3 activation through upstream JAK2 inactivation, reducing STAT3-regulated proteins like Cyclin D1, Bcl-xL, and Mcl-1. This disruption induces apoptosis and reduces tumor growth and angiogenesis in RCC cell lines and mouse models. Additionally, Icaritin modestly affects p-AKT and p-MAPK pathways, further contributing to its anti-tumor effects [61]. Hung et al. found that kaempferol, a natural flavonoid, significantly inhibits the invasion and migration of 786-O RCC cells without cytotoxicity. It lowered MMP-2 expression by inhibiting Akt and FAK phosphorylation, diminishing lung metastasis in SCID mice by up to 87.4%, suggesting its potential in cancer prevention [62]. Bao et al. found that apigenin, a flavonoid with anticancer properties, inhibited RCC cell growth in a concentration-dependent manner. It induced G2/M phase cell cycle arrest, reducing cyclin A, B1, D3, and E levels in both dose- and time-dependent ways, suggesting apigenin’s potential as a therapeutic agent for RCC treatment [63]. Ou et al. found that luteolin sensitized RCC cells to TRAIL-induced apoptosis. While luteolin alone did not affect apoptosis, its combination with TRAIL triggered significant extrinsic and intrinsic apoptosis. This effect was linked to bid cleavage, downregulation of Mcl-1 and FLIP, DR4/DR5 upregulation, and inactivation of Akt and STAT3, highlighting luteolin’s potential as a TRAIL sensitizer in RCC therapy [64]. Nima et al. found that the combination of beta-hydroxybutyrate (BHB) and quercetin (QCT) significantly reduced cell viability in hypoxia-induced Caki-1 cells and downregulated key angiogenesis-related genes and MDR4 expression. The combination therapy notably decreased HIF-1α/2α, VEGF, Ang-1, and MDR4, highlighting its potential to mitigate angiogenesis and drug resistance in RCC treatment [65]. Due to the critical roles that numerous metabolites have in RCC, they are further assessed for their ADMET characteristics and analyzed through molecular docking studies to explore their potential therapeutic applications.

### PPI network analysis of the shortlisted genes

**Fig. 7:**
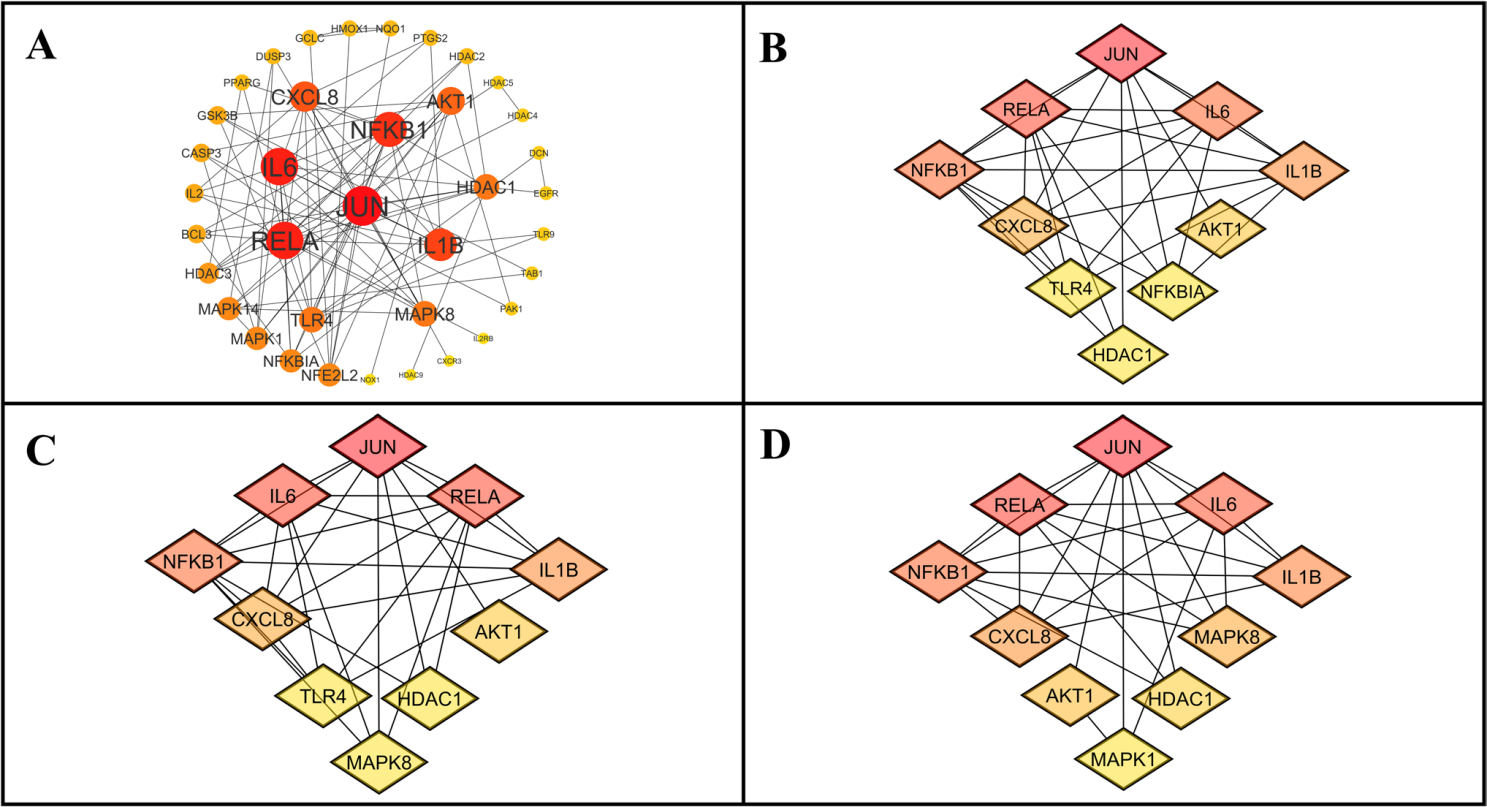
Identification of the hub genes through network analysis **A** PPI analysis of the core target genes highlighting key molecular interactions. **B** MNC Score **C** Degree Score **D** Closeness Score highlighting gene importance in the network

The PPI network analysis of 55 core target genes, illustrated in Fig. 7A, is composed of a network consisting of 37 nodes and 90 edges, providing a foundation for in-depth exploration. Table S5 (Supplementary file) presents the network analysis results, utilizing a confidence interval of 0.900. The top ten hub genes, ranked according to centrality scores from MNC, degree, and closeness, were visualized in Fig. 7B–D to highlight the complexity of the network and reveal key associations, with detailed information provided in Tables S6, S7, and S8 (Supplementary file). Through rigorous analysis, JUN emerged as the prime target gene, with MNC, degree, and closeness scores of 13, 26, and 23.83, respectively, indicating its central role in the network and potential significance in RCC-related gene interactions. JUN, a key member of the transcription factor AP-1, has a critical role in cell proliferation, migration, and invasion. The JUN gene has a significant role in cancer development. c-Jun, a protein from the JUN gene, serves as an oncogene, promoting cancer cell growth, migration, and invasion. It takes part in the progression of various cancers, including melanoma and breast cancer. c-Jun may influence apoptosis through the p53 pathway and is essential for DNA repair. In contrast, JunB, another JUN family member, may oppose c-Jun by inhibiting cell proliferation. However, JunB may aid tumor invasion in certain conditions [66]. The JUN gene family has complex and sometimes opposing roles in cancer progression [67].

Additionally, several genes are reported for their roles in the pathogenesis of RCC. RELA, a subunit of the NF-κB transcription factor, activates pro-inflammatory and anti-apoptotic genes, driving tumor growth, angiogenesis, and therapy resistance [68]. IL6 promotes RCC progression through the JAK/STAT and PI3K/AKT pathways, enhancing angiogenesis and immune evasion, with elevated levels correlating with poor prognosis [69]. NFKB1 regulates inflammation and cell survival by activating genes involved in proliferation, angiogenesis, and metastasis, contributing to therapy resistance [70]. IL1B fosters chronic inflammation, tumor microenvironment remodeling, angiogenesis, and immune suppression, with increased expression linked to RCC aggressiveness [71]. AKT1, a critical component of the PI3K/AKT pathway, supports cell survival, proliferation, and angiogenesis while inhibiting apoptosis, with its dysregulation driving tumor growth and metastasis [72]. CXCL8 (IL-8) modulates the tumor microenvironment, promoting angiogenesis, immune evasion, and metastasis, with high expression levels associated with advanced disease stages and poor outcomes [73]. These genes represent significant therapeutic targets in RCC management.

### ADME/T analysis of the bioactive metabolites

A total of 184 GMMs were initially curated for this investigation. These metabolites underwent a comprehensive screening process, beginning with an evaluation based on Lipinski’s rule of five, as presented in Table S9 (Supplementary file). Metabolites that satisfied Lipinski’s criteria were assessed further, including TPSA (Topological polar surface area), drug likeliness, bioavailability, carcinogenicity, and mutagenicity tests are shown in Table 1. The scrupulous choice of these metabolites set the stage for molecular docking analysis, emphasizing those with favorable pharmacokinetic properties.

**Table 1:** ADME/T profiling of the 28 GMM screened according to Lipinski’s rule

Name of metabolite	PubChem ID	Molecular weight (g/mol)	Bioavailability score	Drug likeliness	TPSA (Å)	Carcinogenicity	Ames toxicity
Nicotinic acid	938	123.11	0.85	0.3	50.19	0.077	0.024
Luteolin	5280445	286.24	0.55	0.38	111.13	0.095	0.536
Apigenin	5280443	270.24	0.55	0.39	90.9	0.277	0.475
6,7,4′-Trihydroxyisoflavone	5284649	270.24	0.55	0.4	90.9	0.403	0.18
Bile acid	439520	408.6	0.56	0.43	97.99	0.019	0.01
Genistein	5280961	270.24	0.55	0.44	90.9	0.316	0.206
4-Pyridoxic acid	6723	183.16	0.56	0.44	90.65	0.053	0.034
Ursodeoxycholic acid	31401	392.6	0.56	0.45	77.76	0.031	0.011
Kaempferol	5280863	286.24	0.55	0.5	111.13	0.097	0.672
Quercetin	5280343	302.23	0.55	0.52	131.36	0.05	0.657
Citric acid	311	192.12	0.56	0.52	132.13	0.009	0.024
3-(3,4-Dihydroxyphenyl)-2-hydroxypropanoic acid	439435	198.17	0.56	0.57	97.99	0.257	0.713
Levodopa	6047	197.19	0.55	0.58	103.78	0.116	0.182
Ponciretin	25201019	285.27	0.56	0.58	78.82	0.652	0.474
Tartaric acid	875	150.09	0.56	0.59	115.06	0.005	0.02
5-(3,4-Dihydroxyphenyl)-valerolactone	45093073	208.21	0.55	0.61	66.76	0.62	0.427
4-Hydroxy-(3′,4′-dihydroxyphenyl)-valeric acid	52920332	226.23	0.56	0.66	97.99	0.12	0.199
Dihydroglycitein	101101166	286.28	0.55	0.79	75.99	0.387	0.543
Dihydrodaidzein	176907	256.25	0.55	0.82	66.76	0.51	0.719
Naringenin	439246	272.25	0.55	0.82	86.99	0.576	0.342
Icaritin	5318980	368.4	0.55	0.84	100.13	0.09	0.701
Dihydrogenistein	9838356	272.25	0.55	0.92	86.99	0.241	0.634
Pioglitazone	4829	356.4	0.55	0.94	93.59	0.345	0.074
6′-Hydroxy-O-desmethylangolensin	20601635	274.27	0.55	1	97.99	0.098	0.66
O-Desmethylangolensin	89472	258.27	0.55	1.2	77.76	0.296	0.554
8-Prenylnaringenin	480764	340.4	0.55	1.36	86.99	0.298	0.134
Deoxycholic acid	222528	392.6	0.56	0.31	77.76	0.022	0.009
11-Methoxycurvularin	10381440	322.4	0.55	0.32	93.06	0.229	0.095

The ADME/T analysis offers a comprehensive evaluation of the drug-likeness and pharmacokinetic characteristics of 28 metabolites, focusing on parameters such as molecular weight, bioavailability, and TPSA. The majority of the compounds exhibit moderate bioavailability scores (0.55–0.85), while some, such as Pioglitazone and O-Desmethylangolensin, demonstrate superior drug-likeness scores, suggesting enhanced potential as therapeutic agents. The TPSA values show considerable variability, with higher values, such as those of Quercetin (131.36 Å^2^), suggesting potential challenges in cellular membrane permeability. Dihydrodaidzein, Naringenin, Icaritin, Dihydrogenistein, Pioglitazone, 6’-Hydroxy-O-desmethylangolensin, O-Desmethylangolensin, and 8-Prenylnaringenin have relatively high drug-likeness value (0.80–1.35), indicating strong potential for therapeutic applications. Carcinogenicity and Ames test results reveal a spectrum of toxicity risks, with most compounds showing low to moderate carcinogenic potential. However, certain metabolites, including 8-Prenylnaringenin and Levodopa, exhibit elevated Ames test scores, which may indicate mutagenic risks. Therefore, these metabolites display a favorable balance of desirable drug properties, though considerations related to bioavailability, permeability, and safety are crucial for further development.

### Molecular docking analysis

**Fig. 8:**
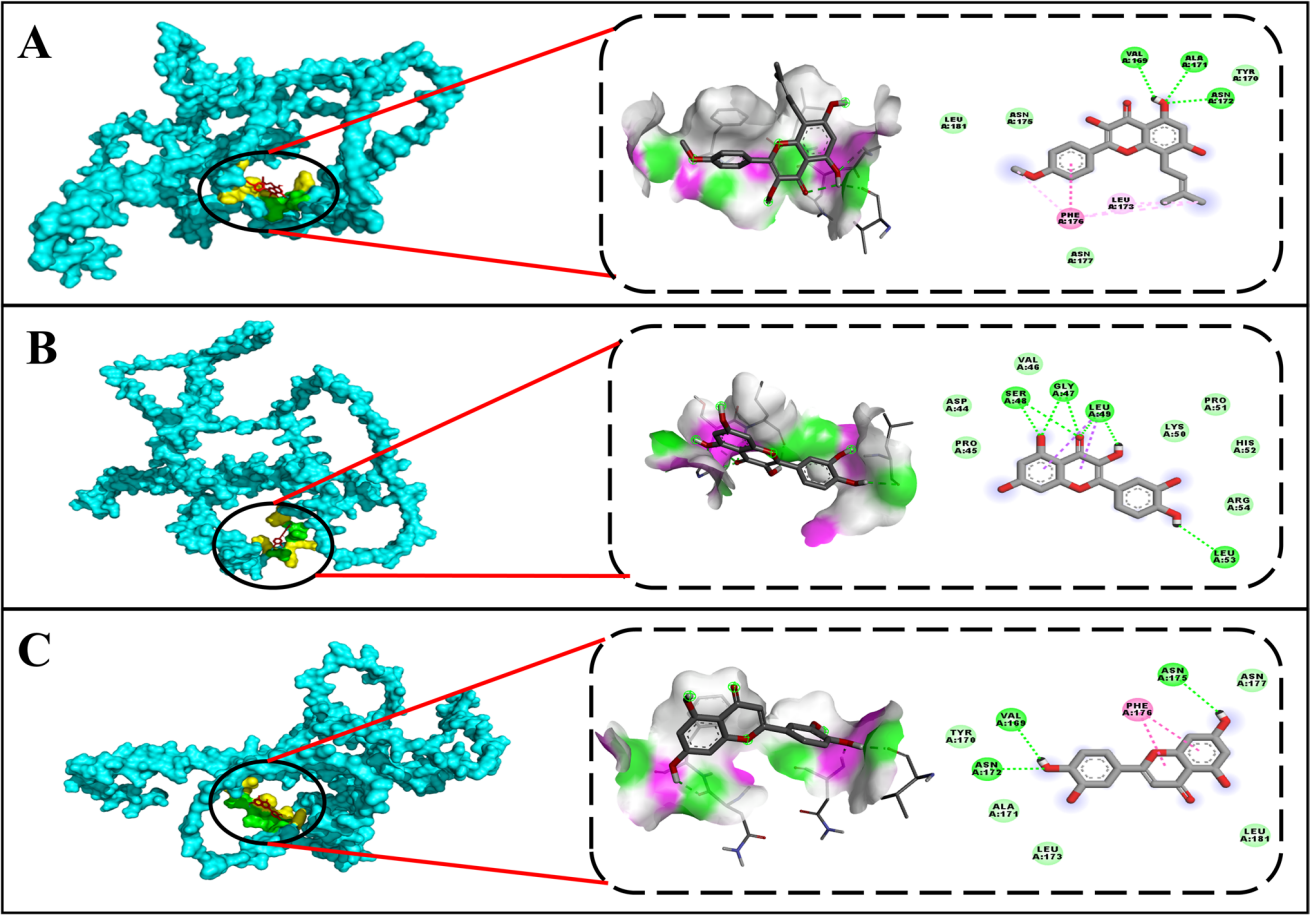
Surface and amino acid interaction analysis of the molecular docking showcasing the detailed binding interactions between **A** JUN and Icaritin. **B** JUN and Quercetin. **C** JUN with Luteolin (Left to right: Red- ligand, Green- polar interactions, Yellow- Other interactions; Purple- H bond donor, Green- H bond acceptor)

The root of the investigation delves into molecular docking analysis, between the identified prime target gene, JUN, and the selected 28 metabolites. The structure of the identified JUN gene was retrieved from AlphaFold (ID: AF-P05412-F1). The grid box coordinates were set to X = 8.056, Y = 6.500, Z = − 34.278, and the dimensions of the grid box were set to X = 126, Y = 126, Z = 126. Table 2 and Table S10 (Supplementary file) offer a comprehensive tabular representation of hydrogen bond interactions and other critical molecular contacts between the 28 ligands and the prime target gene and Fig. 8 illustrates the top three protein–ligand interactions, showcasing the ligand’s interacting sites and the residues within the binding pocket protein. Notably, the highest binding affinity was between Icaritin and JUN as depicted in Fig. 8A, where binding energy of − 5.9 kcal/mol is observed, with a hydrogen bond interaction with VAL169, ALA171, ASN172, Van der Waal interactions with LEU181, ASN175, ASN175, Pi-Pi Stacked interactions with PHE 176, and Pi-Alkyl Interactions with LEU173. Figure 8B illustrates approximately 4 hydrogen bond interactions between JUN and Quercetin, involving GLY47, SER48, LEU49, and LEU53, with a binding energy of − 5.8 kcal/mol. In contrast, there are only 3 hydrogen bond interactions between JUN and Luteolin as demonstrated in Fig. 8C, specifically involving VAL169, ASN172, and ASN175, with a similar binding energy of − 5.8 kcal/mol. The molecular docking analysis provides essential insights into Icaritin’s inhibitory potential against JUN by revealing strong binding interactions and high binding affinity. Icaritin exhibited the highest binding energy (− 5.9 kcal/mol) with JUN, forming stable hydrogen bonds and diverse molecular contacts, including Van der Waals, Pi-Pi, and Pi-Alkyl interactions, especially within the binding pocket. These interactions indicate a firm binding mode, stabilizing JUN in a rigid conformation that may inhibit its typical activity. Comparatively weaker binding affinities and fewer interactions with other metabolites further underscore Icaritin’s unique inhibitory capacity. Thus, molecular docking effectively supports Icaritin’s potential as a JUN inhibitor in computational models.

**Table 2:** Molecular docking analysis of JUN with the top 3 docked complexes

Ligand	PubChem ID	Binding energy (kcal/mol)	H- bond interaction (Amino acid residues)	Other interaction (Amino acid residues)
Icaritin	5318980	− 5.9	VAL169, ALA171, ASN172	TYR170, LEU173, ASN175, PHE176, ASN177, LEU181
Quercetin	5280343	− 5.8	GLY47, SER48, LEU49, LEU53	ASP44, PRO45, VAL46, LYS50, PRO51, HIS52, ARG54
Luteolin	5280445	− 5.8	VAL169, ASN172, ASN175	TYR170, ALA171, LEU173, PHE176, ASN177, LEU181

According to a study conducted by Wu et al., upon consumption of *Epimedium herba*, the flavonoid glycoside icariin undergoes biotransformation in the digestive system through microbial metabolism. Initially, intestinal bacteria such as *Streptococcus* sp. and *Enterococcus* sp. hydrolyze icariin by removing its glucose moiety, resulting in the formation of icariside II. Subsequently, further metabolism by bacteria such as *Blautia* sp. converts icariside II into Icaritin, the aglycone form of icariin, thereby influencing its bioavailability and potential pharmacological effects [74]. Various *in-vitro* studies have demonstrated a wide range of anticancer activity exhibited by Icaritin. It also plays a role in cell-cycle modulation, for example through G_1_ cell cycle arrest and induces down-regulation of phosphorylated pRb, cyclin D1, and CDK4 in human prostate cancer cells. It also shows angiogenesis inhibition wherein CD31 is a marker specifically found on the surface of endothelial cells. In tumor-bearing mice treated with Icariin and Icaritin, there was a significant reduction in CD31-positive areas, suggesting that these compounds exert an anti-hepatoma effect by inhibiting blood vessel formation within the tumor. Other activities include inhibition of metastasis, inhibition of hormone-dependent cancers, inhibition of cancer stem cells, inhibition of cancer cells, and immunomodulatory effect. Icaritin exhibits multiple clinical implications in cancer therapy, including pro-apoptotic activity by increasing the Bax/Bcl-2 ratio, promoting cytochrome c release, inducing poly (ADP-ribose) polymerase (PARP) cleavage, and activating caspases-3, and through the Fas-mediated pathway by upregulating Fas and activating caspase-8 across various cancer types such as the growth of SMMC-7721 hepatoma cells [75]. Additionally, it plays a crucial role in cell cycle regulation, such as inducing G1 cell cycle arrest and downregulating phosphorylated retinoblastoma protein (pRb), cyclin D1, and CDK4 in human prostate cancer cells [76]. Icaritin also demonstrates anti-angiogenic properties, as evidenced by a significant reduction in CD31-positive areas in tumor-bearing mice treated with Icariin and Icaritin, suggesting their potential anti-hepatoma effect through the inhibition of tumor-associated blood vessel formation [77]. Furthermore, Icaritin has been reported to inhibit metastasis [78], suppress hormone-dependent cancers [79] and cancer stem cells [80], inhibition of resistant cancer cells [81], and exert immunomodulatory effects [82], making it a promising candidate for cancer treatment. Several clinical studies have also been conducted to determine the anti-cancerous potentials of Icaritin. A Phase II study (NCT01972672) evaluated Icaritin, an IL-6/STAT3 modulator, in 70 advanced Hepatocellular carcinoma (HCC) patients. Administered 600 mg twice daily, it showed favorable safety (no ≥ grade III events), a median overall survival (OS) of 254 days, and immune-modulation efficacy, especially in PDL1-positive and IL-6/NLR-favorable subgroups, supporting further Phase III trials [83]. A Phase I study (NCT02496949) evaluated Icaritin in advanced HCC patients receiving 600–800 mg twice daily. It demonstrated favorable safety, a clinical benefit rate (CBR) of 46.7% (6.7% partial response, 40% stable disease), and immune-modulating effects. Findings suggest Icaritin’s potential as an oral immunotherapy for advanced HCC [84]. Icaritin can potentially suppress key oncogenic proteins across cancers, modulating PD-L1 expression in HCC to enhance anti-PD-1 therapy effectiveness [85–89]. PD-L1, crucial in RCC for immune evasion and poor prognosis [85], may similarly be influenced by Icaritin. However, this modulation of PD-L1 and IL-6 signaling was not identified in our network pharmacology study, highlighting JUN as the prime target gene and the IL-17 signaling pathway as the key pathway for therapeutic intervention. According to a study by Zhu et al. [90], Icaritin exhibits significant anti-leukemia potential by activating apoptotic pathways involving the Jun gene. In Bcr/Abl + cells, it enhances phospho-JNK and phospho-c-Jun activation, crucial for promoting apoptosis, while downregulating survival pathways, including Jak-2/Stat-3/Akt, in a dose- and time-dependent manner. Additionally, Icaritin suppresses phospho-p38 and phospho-ERK, selectively disrupting leukemogenic signaling. By targeting the constitutive tyrosine kinase activity of Bcr/Abl, which drives chronic myeloid leukemia through pathways like JNK/SAPK [90].

This study is focused on computational analysis and therefore does not include in vitro or in vivo experiments. The absence of these aspects is due to the study’s reliance on computational methods to explore molecular interactions. Therefore, MDS is employed to investigate biomolecular interactions, structure, and dynamics at the atomic level [91]. MDS can predict protein–ligand binding, conformational changes, and interaction energies, providing insights into molecular behavior and mechanisms [91]. This approach reduces the need for costly and time-consuming in vitro and in vivo studies. The findings derived from MDS will aid in interpreting the results and guide further exploration of Icaritin metabolites for their stable binding and activity on the target protein.

### Molecular dynamics simulations

**Fig. 9:**
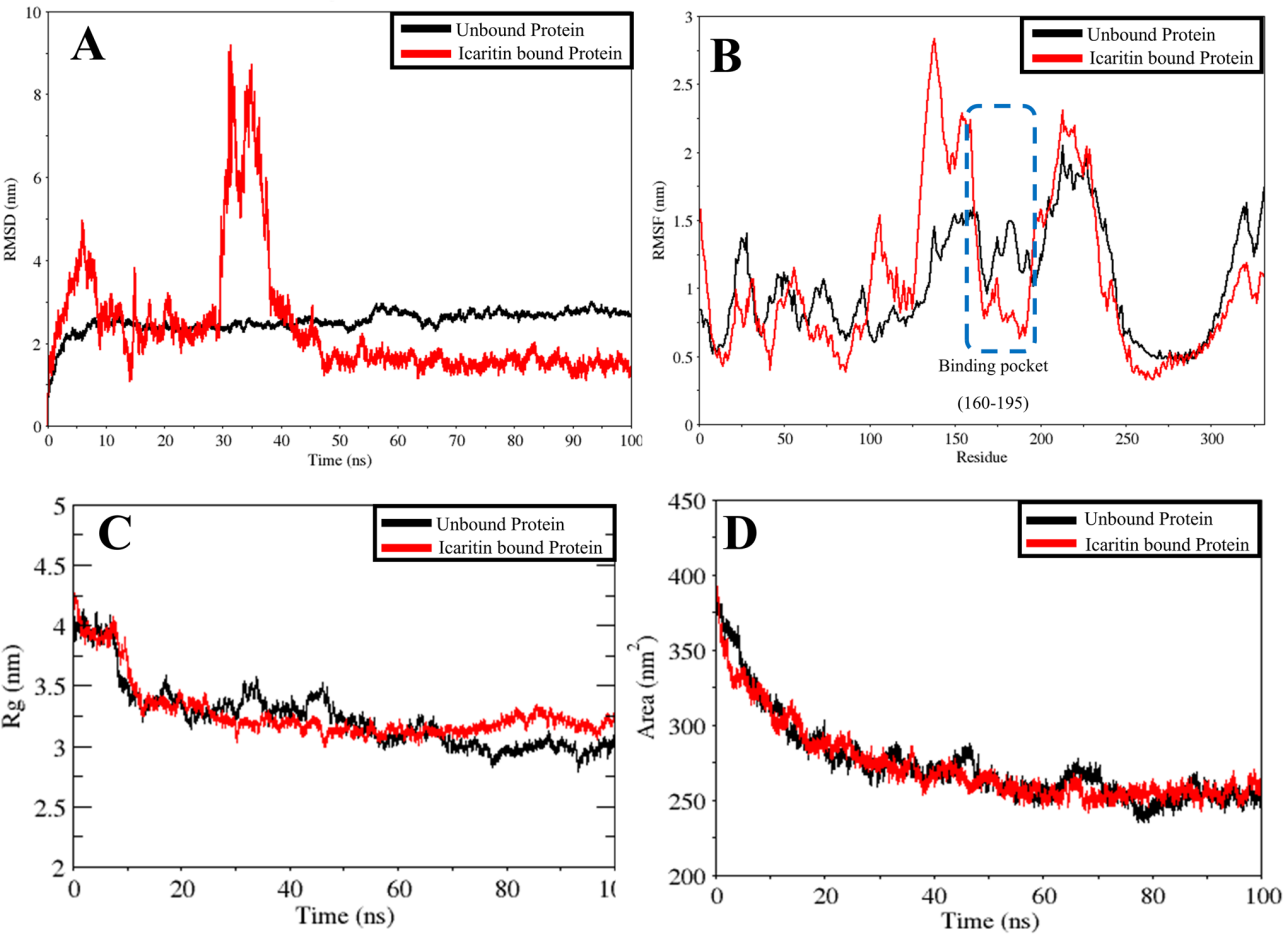
100 ns Molecular Dynamics Simulation Trajectories **A** RMSD **B** RMSF **C** Radius of gyration **D** Surface area plot

The MDS of the complex was carried out to validate the stability of the protein within the biological system. The average RMSD of the unbound JUN was 2.51 nm, indicating an unstable structure. Conversely, when bound with Icaritin, JUN was found to have an average RMSD of 2.34 nm, demonstrating a relatively stable conformation. During the initial 40 ns simulation, JUN displayed high fluctuation, while in the later stages (45–100 ns), a stable conformation was exhibited, as illustrated in Fig. 9A**.** Therefore, Icaritin is believed to adopt a rigid conformation through stable bond formation with JUN.

The RMSF analysis was aligned with the RMSD results, revealing reduced deviation of amino acids from their native states. The unbound protein was found to have a higher amino acid fluctuation of 1.73 nm, contrasting with JUN bound to Icaritin, which showed minimal fluctuation of 1.03 nm. This is illustrated in Fig. 9B, indicating that the average fluctuations of amino acids around the active sites of JUN bound to Icaritin were 0.99 nm, denoting conformational stability compared to the unbound JUN with average fluctuations of 1.25 nm around the active sites. These findings are confirmed to validate the rigidity of Icaritin within the binding pocket throughout the simulation.

The Rg offers insights into the protein’s shape and compactness, revealing the spatial distribution of its constituent atoms. The Rg analysis indicated average values of 2.78 nm for unbound JUN and 3.26 nm for JUN bound with Icaritin, shedding light on the flexibility, and unfolding of the latter. As depicted in Fig. 9C, there was a noticeable decrease in the Rg value of JUN bound with Icaritin from 0 to 50 ns, followed by an increase from 60 to 100 ns. This structural alteration is supported by the RMSD plot of JUN bound with Icaritin, which also demonstrated stability from 50 to 100 ns.

The SASA analysis of unbound JUN revealed a value of 27.78 nm^2^, indicating full accessibility for interactions. In contrast, JUN bound with Icaritin was found to have an average SASA value of 272.64 nm^2^, as illustrated in Fig. 9D. This suggests that the binding of Icaritin to JUN renders it inaccessible, thereby impeding the functions typically demonstrated by JUN.

The MDS indicates that Icaritin binds stably to the JUN protein, significantly reducing its structural fluctuations and rendering it conformationally rigid. The reduction in RMSD and RMSF, along with a high Rg and decreased SASA, highlights that Icaritin binding restricts JUN’s accessibility and flexibility. This rigid conformation likely inhibits JUN’s typical biological functions, supporting the hypothesis that Icaritin can effectively inactivate the JUN gene. Consequently, based on computational analysis, Icaritin’s binding could halt JUN-mediated functions, potentially impeding RCC progression through targeted inhibition of JUN activity.

## Conclusion & Prospects

In the present study, network pharmacology analysis identified the JUN gene as a primary target, with Icaritin—derived from gut microbial metabolite—demonstrating the most potent inhibition binding energy against it. This suggests that targeting the JUN gene, potentially through the IL-17 pathway identified in the enriched pathway analysis and facilitated by Icaritin binding, could offer a promising therapeutic strategy for RCC. It is hypothesized that Icaritin may inhibit the JUN gene, which plays a crucial role in cancer cell growth. This could help slow down tumor growth and offer a new treatment option for RCC. According to a study by Wu et al., the microorganism within the gut involved in producing Icaritin is *Bacterium sp.* MRG-PMF-1. Utilizes Icariin which belongs to the peryl flavones group and is abundantly found in *Epimedii Herba* [74]. Further exploration of Icaritin as an adjunct to conventional therapies, particularly in nanoformulations or as an analog, holds significant promise for RCC treatment. Innovative drug delivery systems, such as nanoencapsulation, can improve bioavailability and enable targeted delivery to cancer cells. Sustained-release formulations and combination therapies with synergistic agents could enhance therapeutic efficacy, reduce dosing frequency, and improve patient adherence. While computational analyses indicate Icaritin’s potential as an anti-RCC agent, rigorous in vitro and in vivo studies are needed for further validation. Its therapeutic promise, demonstrated in clinical investigations, highlights the importance of integrating computational, experimental, and clinical research to comprehensively assess its efficacy.

## Supplementary Information

Below is the link to the electronic supplementary material.

Supplementary file1 (DOCX 63 KB)—The following supporting information can be downloaded at Supplementary Files. Table S1 Comprehensive overview of computational tools and databases employed in the analysis. Table S2 Top 3 gene ontology categories based on fold enrichment scores for cellular components, biological processes, and molecular functions. Table S3 Top 10 significantly enriched pathways identified in the enrichment analysis of RCC. Table S4 Comparative degree values across the M-T-P-D network. Table S5 PPI network analysis of the top 55 genes, highlighting key interactions and their potential implications in the study. Table S6 Top 10 genes identified in the shortest path interaction analysis, ranked according to MNC (Maximum Neighborhood Component) score. Table S7 Top 10 genes identified in the shortest path interaction analysis, ranked according to Degree score. Table S8 Top 10 genes identified in the shortest path interaction analysis, ranked according to Closeness Score. Table S9 Screening of gut microbial metabolites based on Lipinski’s Rule of 5 for drug-likeness assessment. Table S10 Comprehensive molecular docking analysis of JUN protein interactions with the screened gut microbial metabolites.

## Data Availability

Data is provided within the manuscript or supplementary information files.

## Abbreviations

RCC: Renal cell carcinoma
SCFA: Short-chain fatty acids
BCAA: Branched-chain amino acids
ICB: Immune checkpoint blockade
PPI: Protein–protein interaction
GO: Gene ontology
KEGG: Kyoto encyclopedia of genes and genome
MDS: Molecular Dynamics simulations
GMM: Gut microbial metabolites
CRC: Colorectal cancer
SEA: Similarity ensemble approach
STP: Swiss target prediction
OMIM: Online mendelian inheritance in man
FDR: False discovery rate
M-T-P-D: Metabolite- target- pathway- disease
ADME/T: Absorption, distribution, metabolism, excretion, and toxicity
CASTp: Computed Atlas of Surface Topography of Proteins
RMSD: Root mean square deviation
RMSF: Root mean square fluctuations
Rg: Radius of gyration
SASA: Solvent accessible surface area
TPSA: Topological polar surface area
LPS: Lipopolysaccharide
FXR: Farnesoid X receptor
JUN/AP-1: Transcription factor AP-1
IL6: Interleukin 6
RELA: Transcription factor p65
NFKB1: Nuclear factor NF-kappa-B p105 subunit
IL1B: Interleukin-1 beta
CXCL8: Interleukin-8
AKT1: RAC-alpha serine/threonine-protein kinase
TLR4: Toll-like receptor 4
HDAC1: Histone deacetylase 1
MAPK8: Mitogen-activated protein kinase 8
HCC: Hepatocellular carcinoma
OS: Overall survival
CBR: Clinical benefit rate

## References

[1] Bellin M-F, Valente C, Bekdache O, Maxwell F, Balasa C, Savignac A, Meyrignac O (2024) Update on renal cell carcinoma diagnosis with novel imaging approaches. Cancers (Basel). 10.3390/cancers16101926 38792005 10.3390/cancers16101926 PMC11120239

[2] Nakata K, Colombet M, Stiller CA, Pritchard-Jones K, Steliarova-Foucher E (2020) I.-3 contributors, incidence of childhood renal tumours: an international population-based study. Int J Cancer 147:3313–3327. 10.1002/ijc.33147 32902866 10.1002/ijc.33147 PMC7689773

[3] Xu Q, Zhang T, Xia T, Jin B, Chen H, Yang X (2023) Epidemiological trends of kidney cancer along with attributable risk factors in China from 1990 to 2019 and its projections until 2030: an analysis of the global burden of disease study 2019. Clin Epidemiol 15:421–433. 10.2147/CLEP.S400646 37013109 10.2147/CLEP.S400646 PMC10066698

[4] Purdue MP, Dutta D, Machiela MJ, Gorman BR, Winter T, Okuhara D, Cleland S, Ferreiro-Iglesias A, Scheet P, Liu A, Wu C, Antwi SO, Larkin J, Zequi SC, Sun M, Hikino K, Hajiran A, Lawson KA, Cárcano F, Blanchet O, Shuch B, Nepple KG, Margue G, Sundi D, Diver WR, Folgueira MAAK, van Bokhoven A, Neffa F, Brown KM, Hofmann JN, Rhee J, Yeager M, Cole NR, Hicks BD, Manning MR, Hutchinson AA, Rothman N, Huang W-Y, Linehan WM, Lori A, Ferragu M, Zidane-Marinnes M, Serrano SV, Magnabosco WJ, Vilas A, Decia R, Carusso F, Graham LS, Anderson K, Bilen MA, Arciero C, Pellegrin I, Ricard S, Scelo G, Banks RE, Vasudev NS, Soomro N, Stewart GD, Adeyoju A, Bromage S, Hrouda D, Gibbons N, Patel P, Sullivan M, Protheroe A, Nugent FI, Fournier MJ, Zhang X, Martin LJ, Komisarenko M, Eisen T, Cunningham SA, Connolly DC, Uzzo RG, Zaridze D, Mukeria A, Holcatova I, Hornakova A, Foretova L, Janout V, Mates D, Jinga V, Rascu S, Mijuskovic M, Savic S, Milosavljevic S, Gaborieau V, Abedi-Ardekani B, McKay J, Johansson M, Phouthavongsy L, Hayman L, Li J, Lungu I, Bezerra SM, Souza AG, Sares CTG, Reis RB, Gallucci FP, Cordeiro MD, Pomerantz M, Lee G-SM, Freedman ML, Jeong A, Greenberg SE, Sanchez A, Thompson RH, Sharma V, Thiel DD, Ball CT, Abreu D, Lam ET, Nahas WC, Master VA, Patel AV, Bernhard J-C, Freedman ND, Bigot P, Reis RM, Colli LM, Finelli A, Manley BJ, Terao C, Choueiri TK, Carraro DM, Houlston R, Eckel-Passow JE, Abbosh PH, Ganna A, Brennan P, Gu J, Chanock SJ (2024) Multi-ancestry genome-wide association study of kidney cancer identifies 63 susceptibility regions. Nat Genet 56:809–818. 10.1038/s41588-024-01725-7 38671320 10.1038/s41588-024-01725-7 PMC13224750

[5] Motzer RJ, Hutson TE, Cella D, Reeves J, Hawkins R, Guo J, Nathan P, Staehler M, de Souza P, Merchan JR, Boleti E, Fife K, Jin J, Jones R, Uemura H, De Giorgi U, Harmenberg U, Wang J, Sternberg CN, Deen K, McCann L, Hackshaw MD, Crescenzo R, Pandite LN, Choueiri TK (2024) Pazopanib versus sunitinib in metastatic renal-cell carcinoma. N Engl J Med 369:722–731. 10.1056/NEJMoa1303989 10.1056/NEJMoa1303989 23964934

[6] Gabardi S, Baroletti SA (2010) Everolimus: a proliferation signal inhibitor with clinical applications in organ transplantation, oncology, and cardiology. Pharmacother J Hum Pharmacol Drug Ther 30:1044–1056. 10.1592/phco.30.10.1044 10.1592/phco.30.10.1044 20874042

[7] Yang JC, Haworth L, Sherry RM, Hwu P, Schwartzentruber DJ, Topalian SL, Steinberg SM, Chen HX, Rosenberg SA (2024) Randomized trial of bevacizumab, an anti-vascular endothelial growth factor antibody, for metastatic renal cancer. N Engl J Med 349:427–434. 10.1056/NEJMoa021491 10.1056/NEJMoa021491 PMC2275324 12890841

[8] Niewada M, Macioch T, Konarska M, Mela A, Goszczyński A, Przekopińska B, Rajkiewicz K, Wysocki P, Krzakowski M (2023) Immune checkpoint inhibitors combined with tyrosine kinase inhibitors or immunotherapy for treatment-naïve metastatic clear-cell renal cell carcinoma—A network meta-analysis. Focus on cabozantinib combined with nivolumab. Front Pharmacol. 10.3389/fphar.2022.1063178 36937206 10.3389/fphar.2022.1063178 PMC10020696

[9] Schmidinger M, Bellmunt J (2010) Plethora of agents, plethora of targets, plethora of side effects in metastatic renal cell carcinoma. Cancer Treat Rev 36:416–424. 10.1016/j.ctrv.2010.01.003 20163917 10.1016/j.ctrv.2010.01.003

[10] Harmsen HJM, de Goffau MC (2016) The human gut microbiota. In: Schwiertz A (ed) Microbiota of the human body implications in health and disease. Springer, Cham, pp 95–108

[11] Caparrós E, Wiest R, Scharl M, Rogler G, Gutiérrez Casbas A, Yilmaz B, Wawrzyniak M, Francés R (2021) Dysbiotic microbiota interactions in Crohn’s disease. Gut Microbes 13:1949096. 10.1080/19490976.2021.1949096 34313550 10.1080/19490976.2021.1949096 PMC8320851

[12] Del Chierico F, Rapini N, Deodati A, Matteoli MC, Cianfarani S, Putignani L (2022) Pathophysiology of type 1 diabetes and gut microbiota role. Int J Mol Sci. 10.3390/ijms232314650 36498975 10.3390/ijms232314650 PMC9737253

[13] Debédat J, Clément K, Aron-Wisnewsky J (2019) Gut microbiota dysbiosis in human obesity: impact of bariatric surgery. Curr Obes Rep 8:229–242. 10.1007/s13679-019-00351-3 31197613 10.1007/s13679-019-00351-3

[14] Liu J, Tan Y, Cheng H, Zhang D, Feng W, Peng C (2022) Functions of gut microbiota metabolites, current status and future perspectives. Aging Dis 13:1106–1126. 10.14336/AD.2022.0104 35855347 10.14336/AD.2022.0104 PMC9286904

[15] Canfora EE, Meex RCR, Venema K, Blaak EE (2019) Gut microbial metabolites in obesity, NAFLD and T2DM. Nat Rev Endocrinol 15:261–273. 10.1038/s41574-019-0156-z 30670819 10.1038/s41574-019-0156-z

[16] Mokdad AH, Ford ES, Bowman BA, Dietz WH, Vinicor F, Bales VS, Marks JS (2003) Prevalence of obesity, diabetes, and obesity-related health risk factors, 2001. JAMA 289:76–79. 10.1001/jama.289.1.76 12503980 10.1001/jama.289.1.76

[17] Zhu Y, Shui X, Liang Z, Huang Z, Qi Y, He Y, Chen C, Luo H, Lei W (2020) Gut microbiota metabolites as integral mediators in cardiovascular diseases (Review). Int J Mol Med 46:936–948. 10.3892/ijmm.2020.4674 32705240 10.3892/ijmm.2020.4674 PMC7388831

[18] Ahn J, Sinha R, Pei Z, Dominianni C, Wu J, Shi J, Goedert JJ, Hayes RB, Yang L (2013) Human gut microbiome and risk for colorectal cancer. J Natl Cancer Inst 105:1907–1911. 10.1093/jnci/djt300 24316595 10.1093/jnci/djt300 PMC3866154

[19] Wei Z, Cao S, Liu S, Yao Z, Sun T, Li Y, Li J, Zhang D, Zhou Y (2016) Could gut microbiota serve as prognostic biomarker associated with colorectal cancer patients’ survival a pilot study on relevant mechanism. Oncotarget 7:46158–46172. 10.18632/oncotarget.10064 27323816 10.18632/oncotarget.10064 PMC5216788

[20] Kwong TNY, Wang X, Nakatsu G, Chow TC, Tipoe T, Dai RZW, Tsoi KKK, Wong MCS, Tse G, Chan MTV, Chan FKL, Ng SC, Wu JCY, Wu WKK, Yu J, Sung JJY, Wong SH (2018) Association between bacteremia from specific microbes and subsequent diagnosis of colorectal cancer. Gastroenterology 155:383-390.e8. 10.1053/j.gastro.2018.04.028 29729257 10.1053/j.gastro.2018.04.028

[21] Dizman N, Hsu J, Bergerot PG, Gillece JD, Folkerts M, Reining L, Trent J, Highlander SK, Pal SK (2021) Randomized trial assessing impact of probiotic supplementation on gut microbiome and clinical outcome from targeted therapy in metastatic renal cell carcinoma. Cancer Med 10:79–86. 10.1002/cam4.3569 33135866 10.1002/cam4.3569 PMC7826461

[22] Wong CC, Yu J (2023) Gut microbiota in colorectal cancer development and therapy. Nat Rev Clin Oncol 20:429–452. 10.1038/s41571-023-00766-x 37169888 10.1038/s41571-023-00766-x

[23] Liu L, Li M, Yu M, Shen M, Wang Q, Yu Y, Xie J (2019) Natural polysaccharides exhibit anti-tumor activity by targeting gut microbiota. Int J Biol Macromol 121:743–751. 10.1016/j.ijbiomac.2018.10.083 30342142 10.1016/j.ijbiomac.2018.10.083

[24] de Moreno A, de Leblanc G, Perdigón, (2010) The application of probiotic fermented milks in cancer and intestinal inflammation. Proc Nutr Soc 69:421–428. 10.1017/S002966511000159X 20550747 10.1017/S002966511000159X

[25] Derosa L, Routy B, Fidelle M, Iebba V, Alla L, Pasolli E, Segata N, Desnoyer A, Pietrantonio F, Ferrere G, Fahrner J-E, Le Chatellier E, Pons N, Galleron N, Roume H, Duong CPM, Mondragón L, Iribarren K, Bonvalet M, Terrisse S, Rauber C, Goubet A-G, Daillère R, Lemaitre F, Reni A, Casu B, Alou MT, Costa Silva CA, Raoult D, Fizazi K, Escudier B, Kroemer G, Albiges L, Zitvogel L (2020) Gut bacteria composition drives primary resistance to cancer immunotherapy in renal cell carcinoma patients. Eur Urol 78:195–206. 10.1016/j.eururo.2020.04.044 32376136 10.1016/j.eururo.2020.04.044

[26] Salgia NJ, Bergerot PG, Maia MC, Dizman N, Hsu J, Gillece JD, Folkerts M, Reining L, Trent J, Highlander SK, Pal SK (2020) Stool microbiome profiling of patients with metastatic renal cell carcinoma receiving anti–PD-1 immune checkpoint inhibitors. Eur Urol 78:498–502. 10.1016/j.eururo.2020.07.011 32828600 10.1016/j.eururo.2020.07.011

[27] Dizman N, Meza L, Bergerot P, Alcantara M, Dorff T, Lyou Y, Frankel P, Cui Y, Mira V, Llamas M, Hsu J, Zengin Z, Salgia N, Salgia S, Malhotra J, Chawla N, Chehrazi-Raffle A, Muddasani R, Gillece J, Reining L, Trent J, Takahashi M, Oka K, Higashi S, Kortylewski M, Highlander SK, Pal SK (2022) Nivolumab plus ipilimumab with or without live bacterial supplementation in metastatic renal cell carcinoma: a randomized phase 1 trial. Nat Med 28:704–712. 10.1038/s41591-022-01694-6 35228755 10.1038/s41591-022-01694-6 PMC9018425

[28] Cheng L, Qi C, Yang H, Lu M, Cai Y, Fu T, Ren J, Jin Q, Zhang X (2022) gutMGene: a comprehensive database for target genes of gut microbes and microbial metabolites. Nucleic Acids Res 50:D795–D800. 10.1093/NAR/GKAB786 34500458 10.1093/nar/gkab786 PMC8728193

[29] Keiser MJ, Roth BL, Armbruster BN, Ernsberger P, Irwin JJ, Shoichet BK (2007) Relating protein pharmacology by ligand chemistry. Nat Biotechnol 25:197–206. 10.1038/nbt1284 17287757 10.1038/nbt1284

[30] Daina A, Michielin O, Zoete V (2019) SwissTargetPrediction: updated data and new features for efficient prediction of protein targets of small molecules. Nucleic Acids Res 47:W357–W364. 10.1093/nar/gkz382 31106366 10.1093/nar/gkz382 PMC6602486

[31] Heberle H, Meirelles GV, da Silva FR, Telles GP, Minghim R (2015) InteractiVenn: a web-based tool for the analysis of sets through Venn diagrams. BMC Bioinform 16:169. 10.1186/s12859-015-0611-3 10.1186/s12859-015-0611-3 PMC4455604 25994840

[32] Piñero J, Bravo Á, Queralt-Rosinach N, Gutiérrez-Sacristán A, Deu-Pons J, Centeno E, García-García J, Sanz F, Furlong LI (2017) DisGeNET: a comprehensive platform integrating information on human disease-associated genes and variants. Nucleic Acids Res 45:D833–D839. 10.1093/NAR/GKW943 27924018 10.1093/nar/gkw943 PMC5210640

[33] Stelzer G, Rosen N, Plaschkes I, Zimmerman S, Twik M, Fishilevich S, Iny Stein T, Nudel R, Lieder I, Mazor Y, Kaplan S, Dahary D, Warshawsky D, Guan-Golan Y, Kohn A, Rappaport N, Safran M (2016) Lancet, the genecards suite: from gene data mining to disease genome sequence analyses. Curr Protoc Bioinform 54:1301–13033. 10.1002/CPBI.5 10.1002/cpbi.5 27322403

[34] Cohen R, Gefen A, Elhadad M, Birk OS (2011) CSI-OMIM–clinical synopsis search in OMIM. BMC Bioinform. 10.1186/1471-2105-12-65 10.1186/1471-2105-12-65 PMC3053257 21362185

[35] Ashburner M, Ball CA, Blake JA, Botstein D, Butler H, Cherry JM, Davis AP, Dolinski K, Dwight SS, Eppig JT, Harris MA, Hill DP, Issel-Tarver L, Kasarskis A, Lewis S, Matese JC, Richardson JE, Ringwald M, Rubin GM, Sherlock G (2000) Gene Ontology: tool for the unification of biology. Nat Genet 25:25–29. 10.1038/75556 10802651 10.1038/75556 PMC3037419

[36] Ge SX, Jung D, Jung D, Yao R (2020) ShinyGO: a graphical gene-set enrichment tool for animals and plants. Bioinformatics 36:2628–2629. 10.1093/BIOINFORMATICS/BTZ931 31882993 10.1093/bioinformatics/btz931 PMC7178415

[37] Kanehisa M, Furumichi M, Sato Y, Ishiguro-Watanabe M, Tanabe M (2020) KEGG: integrating viruses and cellular organisms. Nucleic Acids Res 49:D545–D551. 10.1093/nar/gkaa970 10.1093/nar/gkaa970 PMC7779016 33125081

[38] Shannon P, Markiel A, Ozier O, Baliga NS, Wang JT, Ramage D, Amin N, Schwikowski B, Ideker T (2003) Cytoscape: a software environment for integrated models of biomolecular interaction networks. Genome Res 13:2498–2504. 10.1101/gr.1239303 14597658 10.1101/gr.1239303 PMC403769

[39] Luo W, Brouwer C (2013) Pathview: an R/Bioconductor package for pathway-based data integration and visualization. Bioinformatics 29:1830–1831. 10.1093/bioinformatics/btt285 23740750 10.1093/bioinformatics/btt285 PMC3702256

[40] Chen B, Fan W, Liu J, Wu F-X (2013) Identifying protein complexes and functional modules—from static PPI networks to dynamic PPI networks. Brief Bioinform 15:177–194. 10.1093/bib/bbt039 23780996 10.1093/bib/bbt039

[41] Doncheva NT, Morris JH, Gorodkin J, Jensen LJ (2019) Cytoscape stringapp: network analysis and visualization of proteomics data. J Proteome Res 18:623–632. 10.1021/ACS.JPROTEOME.8B00702 30450911 10.1021/acs.jproteome.8b00702 PMC6800166

[42] Chin C-H, Chen S-H, Wu H-H, Ho C-W, Ko M-T, Lin C-Y (2014) cytoHubba: identifying hub objects and sub-networks from complex interactome. BMC Syst Biol 8:S11. 10.1186/1752-0509-8-S4-S11 25521941 10.1186/1752-0509-8-S4-S11 PMC4290687

[43] Yip AM, Horvath S (2007) Gene network interconnectedness and the generalized topological overlap measure. BMC Bioinform 8:22. 10.1186/1471-2105-8-22 10.1186/1471-2105-8-22 PMC1797055 17250769

[44] Wang Y, Xing J, Xu Y, Zhou N, Peng J, Xiong Z, Liu X, Luo X, Luo C, Chen K, Zheng M, Jiang H (2015) In silico ADME/T modelling for rational drug design. Q Rev Biophys 48:488–515. 10.1017/S0033583515000190 26328949 10.1017/S0033583515000190

[45] Daina A, Michielin O, Zoete V (2017) SwissADME: a free web tool to evaluate pharmacokinetics, drug-likeness and medicinal chemistry friendliness of small molecules. Sci Rep. 10.1038/SREP42717 28256516 10.1038/srep42717 PMC5335600

[46] Xiong G, Wu Z, Yi J, Fu L, Yang Z, Hsieh C, Yin M, Zeng X, Wu C, Lu A, Chen X, Hou T, Cao D (2021) ADMETlab 2.0: an integrated online platform for accurate and comprehensive predictions of ADMET properties. Nucleic Acids Res 49:W5–W14. 10.1093/NAR/GKAB255 33893803 10.1093/nar/gkab255 PMC8262709

[47] Chagas CM, Moss S, Alisaraie L (2018) Drug metabolites and their effects on the development of adverse reactions: revisiting Lipinski’s rule of five. Int J Pharm 549:133–149. 10.1016/j.ijpharm.2018.07.046 30040971 10.1016/j.ijpharm.2018.07.046

[48] Tian W, Chen C, Lei X, Zhao J, Liang J (2018) CASTp 3.0: computed atlas of surface topography of proteins. Nucleic Acids Res 46:W363–W367. 10.1093/NAR/GKY473 29860391 10.1093/nar/gky473 PMC6031066

[49] Tang S, Chen R, Lin M, Lin Q, Zhu Y, Ding J, Hu H, Ling M, Wu J (2022) Accelerating autodock vina with GPUs. Molecules. 10.3390/MOLECULES27093041 35566391 10.3390/molecules27093041 PMC9103882

[50] Rosignoli S, Paiardini A (2022) Boosting the full potential of PyMOL with structural biology plugins. Biomolecules. 10.3390/BIOM12121764 36551192 10.3390/biom12121764 PMC9775141

[51] Karplus M, Petsko GA (1990) Molecular dynamics simulations in biology. Nature 347:631–639. 10.1038/347631a0 2215695 10.1038/347631a0

[52] Stany B, Mishra S, Rao KVB (2024) Pharmacokinetic studies, molecular docking, and molecular dynamics simulations of phytochemicals from Morus alba: a multi receptor approach for potential therapeutic agents in colorectal cancer. Med Oncol 41:156. 10.1007/s12032-024-02406-5 38750377 10.1007/s12032-024-02406-5

[53] Nag S, Stany B, Mishra S, Kumar S, Mohanto S, Ahmed MG, Mathew B, Subramaniyan V (2024) Multireceptor analysis for evaluating the antidiabetic efficacy of Karanjin: a computational approach. Endocrinol Diabetes Metab 7:e509. 10.1002/edm2.509 38982323 10.1002/edm2.509 PMC11233261

[54] Bansal N, David G, Farias E, Waxman S (2016) Emerging roles of epigenetic regulator Sin3 in cancer. Adv Cancer Res 130:113–135. 10.1016/bs.acr.2016.01.006 27037752 10.1016/bs.acr.2016.01.006

[55] Brito GC, Fachel ÂA, Vettore AL, Vignal GM, Gimba ERP, Campos FS, Barcinski MA, Verjovski-Almeida S, Reis EM (2008) Identification of protein-coding and intronic noncoding RNAs down-regulated in clear cell renal carcinoma. Mol Carcinog 47:757–767. 10.1002/mc.20433 18348187 10.1002/mc.20433

[56] Yang Y, Yang L, Jiang S, Yang T, Lan J, Lei Y, Tan H, Pan K (2020) HMGB1 mediates lipopolysaccharide-induced inflammation via interacting with GPX4 in colon cancer cells. Cancer Cell Int 20:205. 10.1186/s12935-020-01289-6 32514250 10.1186/s12935-020-01289-6 PMC7260829

[57] Nenkov M, Shi Y, Ma Y, Gaßler N, Chen Y (2024) Targeting farnesoid x receptor in tumor and the tumor microenvironment: implication for therapy. Int J Mol Sci. 10.3390/ijms25010006 38203175 10.3390/ijms25010006 PMC10778939

[58] McGeachy MJ, Cua DJ, Gaffen SL (2019) The IL-17 family of cytokines in health and disease. Immunity 50:892–906. 10.1016/j.immuni.2019.03.021 30995505 10.1016/j.immuni.2019.03.021 PMC6474359

[59] Jarocki M, Karska J, Kowalski S, Kiełb P, Nowak Ł, Krajewski W, Saczko J, Kulbacka J, Szydełko T, Małkiewicz B (2022) Interleukin 17 and its involvement in renal cell carcinoma. J Clin Med. 10.3390/jcm11174973 36078902 10.3390/jcm11174973 PMC9457171

[60] Zenobia C, Hajishengallis G (2015) Basic biology and role of interleukin-17 in immunity and inflammation. Periodontol 2000(69):142. 10.1111/PRD.12083 10.1111/prd.12083 PMC4530463 26252407

[61] Li S, Priceman SJ, Xin H, Zhang W, Deng J, Liu Y, Huang J, Zhu W, Chen M, Hu W, Deng X, Zhang J, Yu H, He G (2013) Icaritin inhibits JAK/STAT3 signaling and growth of renal cell carcinoma. PLoS ONE 8:1–8. 10.1371/journal.pone.0081657 10.1371/journal.pone.0081657 PMC3855768 24324713

[62] Hung T-W, Chen P-N, Wu H-C, Wu S-W, Tsai P-Y, Hsieh Y-S, Chang H-R (2017) Kaempferol inhibits the invasion and migration of renal cancer cells through the downregulation of AKT and FAK pathways. Int J Med Sci 14:984–993. 10.7150/ijms.20336 28924370 10.7150/ijms.20336 PMC5599922

[63] Bao Y, Wu X, Jin X, Kanematsu A, Nojima M, Kakehi Y, Yamamoto S (2022) Apigenin inhibits renal cell carcinoma cell proliferation through G2/M phase cell cycle arrest. Oncol Rep 47:60. 10.3892/or.2022.8271 35088891 10.3892/or.2022.8271

[64] Ou Y-C, Li J-R, Kuan Y-H, Raung S-L, Wang C-C, Hung Y-Y, Pan P-H, Lu H-C, Chen C-J (2014) Luteolin sensitizes human 786-O renal cell carcinoma cells to TRAIL-induced apoptosis. Life Sci 100:110–117. 10.1016/j.lfs.2014.02.002 24530290 10.1016/j.lfs.2014.02.002

[65] Mohammadipoor N, Naiebi R, Mazhari SA, Amooei F, Owrang M, Dastghaib S, Shams M, Maleki MH, Dastghaib S (2024) Improved therapy for clear cell renal cell carcinoma: beta-hydroxybutyrate and quercetin target hypoxia-induced angiogenesis and multidrug resistance. Mol Biol Rep 51:379. 10.1007/s11033-024-09355-2 38429605 10.1007/s11033-024-09355-2

[66] Koo AS, Chiu R, Soong J, Dekernion JB, Belldegrun A (1992) The expression of C-jun and junB mRNA in renal cell cancer and in vitro regulation by transforming growth factor beta 1 and tumor necrosis factor alpha 1. J Urol 148:1314–1318. 10.1016/s0022-5347(17)36899-4 1404666 10.1016/s0022-5347(17)36899-4

[67] Song D, Lian Y, Zhang L (2023) The potential of activator protein 1 (AP-1) in cancer targeted therapy. Front Immunol. 10.3389/fimmu.2023.1224892 37483616 10.3389/fimmu.2023.1224892 PMC10361657

[68] Hwa JS, Kim FJ, Kumar B, Koul S, Meacham R, Koul H (2010) Curcumin targets RELA (P65) stability to inhibit constitutive NF-KAPPA B activation and induces apoptosis in human renal cell carcinoma. J Urol 75:183

[69] Koo AS, Armstrong C, Bochner B, Shimabukuro T, Tso C-L, DeKernion JB, Belldegrun A (1992) Interleukin-6 and renal cell cancer: production, regulation, and growth effects. Cancer Immunol Immunother 35:97–105. 10.1007/BF01741856 1596939 10.1007/BF01741856 PMC11037957

[70] Sun J, Chen F, Wu G (2023) Role of NF-κB pathway in kidney renal clear cell carcinoma and its potential therapeutic implications. Aging. 10.18632/aging.205129 37847185 10.18632/aging.205129 PMC10637793

[71] Aggen DH, Ager CR, Obradovic AZ, Chowdhury N, Ghasemzadeh A, Mao W, Chaimowitz MG, Lopez-Bujanda ZA, Spina CS, Hawley JE, Dallos MC, Zhang C, Wang V, Li H, Guo XV, Drake CG (2021) Blocking IL1 beta promotes tumor regression and remodeling of the myeloid compartment in a renal cell carcinoma model: multidimensional analyses. Clin Cancer Res 27:608–621. 10.1158/1078-0432.CCR-20-1610 33148676 10.1158/1078-0432.CCR-20-1610 PMC7980495

[72] Fan D, Liu Q, Wu F, Liu N, Qu H, Yuan Y, Li Y, Gao H, Ge J, Xu Y, Wang H, Liu Q, Zhao Z (2020) Prognostic significance of PI3K/AKT/ mTOR signaling pathway members in clear cell renal cell carcinoma. PeerJ 8:e9261. 10.7717/peerj.9261 32547875 10.7717/peerj.9261 PMC7271881

[73] Shen J, Wang R, Chen Y, Fang Z, Tang J, Yao J, Gao J, Chen X, Shi X (2023) Prognostic significance and mechanisms of CXCL genes in clear cell renal cell carcinoma. Aging 15:7974–7996. 10.18632/aging.204922 37540227 10.18632/aging.204922 PMC10497021

[74] Wu H, Kim M, Han J (2016) Icariin metabolism by human intestinal microflora. Molecules. 10.3390/molecules21091158 27589718 10.3390/molecules21091158 PMC6273050

[75] Sun L, Peng Q, Qu L, Gong L, Si J (2015) Anticancer agent icaritin induces apoptosis through caspase-dependent pathways in human hepatocellular carcinoma cells. Mol Med Rep 11:3094–3100. 10.3892/mmr.2014.3007 25434584 10.3892/mmr.2014.3007

[76] Huang X, Zhu D, Lou Y (2007) A novel anticancer agent, icaritin, induced cell growth inhibition, G1 arrest and mitochondrial transmembrane potential drop in human prostate carcinoma PC-3 cells. Eur J Pharmacol 564:26–36. 10.1016/j.ejphar.2007.02.039 17382317 10.1016/j.ejphar.2007.02.039

[77] Li Q, Huai L, Zhang C, Wang C, Jia Y, Chen Y, Yu P, Wang H, Rao Q, Wang M, Wang J (2013) Icaritin induces AML cell apoptosis via the MAPK/ERK and PI3K/AKT signal pathways. Int J Hematol 97:617–623. 10.1007/s12185-013-1317-9 23550021 10.1007/s12185-013-1317-9

[78] Xu B, Jiang C, Han H, Liu H, Tang M, Liu L, Ji W, Lu X, Yang X, Zhang Y, Liu Y (2015) Icaritin inhibits the invasion and epithelial-to-mesenchymal transition of glioblastoma cells by targeting EMMPRIN via PTEN/AKt/HIF-1 α signalling. Clin Exp Pharmacol Physiol 42:1296–1307. 10.1111/1440-1681.12488 26356761 10.1111/1440-1681.12488

[79] Indran I, Zhang S-J, Zhang Z, Sun F, Gong Y, Wang X, Li J, Erdelmeier C, Koch E, Yong E (2013) Selective estrogen receptor modulator effects of epimedium extracts on breast cancer and uterine growth in nude mice. Planta Med 80:22–28. 10.1055/s-0033-1360112 24310211 10.1055/s-0033-1360112

[80] Zhao H (2015) 225P A novel anti-cancer agent icaritin suppresses hepatocellular carcinoma initiation and malignant growth through the IL-6/Jak2/Stat3 pathway. Ann Oncol 26:ix42. 10.1093/annonc/mdv523.86 10.18632/oncotarget.5578 PMC4741651 26376676

[81] Sun L, Chen W, Qu L, Wu J, Si J (2013) Icaritin reverses multidrug resistance of HepG2/ADR human hepatoma cells via downregulation of MDR1 and P-glycoprotein expression. Mol Med Rep 8:1883–1887. 10.3892/mmr.2013.1742 24145579 10.3892/mmr.2013.1742

[82] Zhou J, Wu J, Chen X, Fortenbery N, Eksioglu E, Kodumudi KN, PK EB, Dong J, Djeu JY, Wei S (2011) Icariin and its derivative, ICT, exert anti-inflammatory, anti-tumor effects, and modulate myeloid derived suppressive cells (MDSCs) functions. Int Immunopharmacol 11:890–898. 10.1016/j.intimp.2011.01.007 21244860 10.1016/j.intimp.2011.01.007 PMC3109231

[83] Sun Y, Li Q, Xu J-M, Liang J, Cheng Y, Li S, Zheng L, Ye B, Meng K, Qin S (2018) A multicenter, single arm phase II trial of a small molecule immune-modulator icaritin: Safety, overall survival, immune dynamics, and PD-L1 expression in advanced hepatocellular carcinoma. J Clin Oncol 36:4077–4077. 10.1200/JCO.2018.36.15_suppl.4077

[84] Fan Y, Li S, Ding X, Yue J, Jiang J, Zhao H, Hao R, Qiu W, Liu K, Li Y, Wang S, Zheng L, Ye B, Meng K, Xu B (2019) First-in-class immune-modulating small molecule Icaritin in advanced hepatocellular carcinoma: preliminary results of safety, durable survival and immune biomarkers. BMC Cancer 19:279. 10.1186/s12885-019-5471-1 30922248 10.1186/s12885-019-5471-1 PMC6437929

[85] Kumar B, Ghosh A, Datta C, Pal DK (2019) Role of PDL1 as a prognostic marker in renal cell carcinoma: a prospective observational study in eastern India. Ther Adv Urol. 10.1177/1756287219868859 31447938 10.1177/1756287219868859 PMC6691662

[86] Tao H, Liu M, Wang Y, Luo S, Xu Y, Ye B, Zheng L, Meng K, Li L (2021) Icaritin induces anti-tumor immune responses in hepatocellular carcinoma by inhibiting splenic myeloid-derived suppressor cell generation. Front Immunol. 10.3389/fimmu.2021.609295 33717093 10.3389/fimmu.2021.609295 PMC7952329

[87] Hao H, Zhang Q, Zhu H, Wen Y, Qiu D, Xiong J, Fu X, Wu Y, Meng K, Li J (2019) Icaritin promotes tumor T-cell infiltration and induces antitumor immunity in mice. Eur J Immunol 49:2235–2244. 10.1002/eji.201948225 31465113 10.1002/eji.201948225

[88] Dongye Z, Wu X, Wen Y, Ding X, Wang C, Zhao T, Li J, Wu Y (2022) Icaritin and intratumoral injection of CpG treatment synergistically promote T cell infiltration and antitumor immune response in mice. Int Immunopharmacol 111:109093. 10.1016/j.intimp.2022.109093 35930912 10.1016/j.intimp.2022.109093

[89] Mo D, Zhu H, Wang J, Hao H, Guo Y, Wang J, Han X, Zou L, Li Z, Yao H, Zhu J, Zhou J, Peng Y, Li J, Meng K (2021) Icaritin inhibits PD-L1 expression by targeting protein IκB kinase α. Eur J Immunol 51:978–988. 10.1002/eji.202048905 33354776 10.1002/eji.202048905 PMC8248075

[90] Feng Zhu J, Jian Li Z, Sen Zhang G, Meng K, Yong Kuang W, Li J, Fu Zhou X, Juan Li R, Ling Peng H, Wen Dai C, Shen JK, Jie Gong F, Xiao Xu Y, Fang Liu S (2011) Icaritin shows potent anti-leukemia activity on chronic myeloid leukemia in vitro and in vivo by regulating MAPK/ERK/JNK and JAK2/STAT3/AKT signalings. PLoS ONE 6:e23720. 10.1371/journal.pone.0023720 21887305 10.1371/journal.pone.0023720 PMC3161749

[91] Hollingsworth SA, Dror RO (2018) Molecular dynamics simulation for All. Neuron 99:1129–1143. 10.1016/j.neuron.2018.08.011 30236283 10.1016/j.neuron.2018.08.011 PMC6209097

