# Supplementary material for: Gut microbial metabolites targeting JUN in renal cell carcinoma via IL-17 signaling pathway: network pharmacology approach
Source: Mol Divers. 2025 Apr 18;30(1):613–32. doi: 10.1007/s11030-025-11188-5 (PMC12926257; doi:10.1007/s11030-025-11188-5)
Supplement: Supplementary file 1 — Supplementary file1 (DOCX 63 KB)—The following supporting information can be downloaded at Supplementary Files. Table S1 Comprehensive overview of computational tools and databases employed in the analysis. Table S2 Top 3 gene ontology categories based on fold enrichment scores for cellular components, biological processes, and molecular functions. Table S3 Top 10 significantly enriched pathways identified in the enrichment analysis of RCC. Table S4 Comparative degree values across the M-T-P-D network. Table S5 PPI network analysis of the top 55 genes, highlighting key interactions and their potential implications in the study. Table S6 Top 10 genes identified in the shortest path interaction analysis, ranked according to MNC (Maximum Neighborhood Component) score. Table S7 Top 10 genes identified in the shortest path interaction analysis, ranked according to Degree score. Table S8 Top 10 genes identified in the shortest path interaction analysis, ranked according to Closeness Score. Table S9 Screening of gut microbial metabolites based on Lipinski’s Rule of 5 for drug-likeness assessment. Table S10 Comprehensive molecular docking analysis of JUN protein interactions with the screened gut microbial metabolites. [file 11030_2025_11188_MOESM1_ESM.docx]

**Gut Microbial Metabolites Targeting JUN in Renal Cell Carcinoma via IL-17 Signaling Pathway: Network Pharmacology Approach**

B Stany ^#1^, Shatakshi Mishra ^#1^, Anushka Das ^1^, Sagnik Nag^2^, Rakesh Naidu^2^

**Supplementary Material**

**Table S1** Comprehensive overview of computational tools and databases employed in the analysis

| **Software/ Database** | **Description** |
| --- | --- |
| GutMgene (<http://bio-annotation.cn/gutmgene/browse.dhtml>) | Extraction of human gut microbial fermented metabolites and genes |
| Similarity ensemble approach (<https://sea.bkslab.org/>)  Swiss Target Prediction (<http://www.swisstargetprediction.ch/>) | Extraction of genes for the metabolites |
| DisGeNET (<https://www.disgenet.org/search>)  GeneCards (<https://www.genecards.org/>)  OMIM (<https://www.omim.org/>) | Extraction of RCC genes |
| InteractiVenn (<https://www.interactivenn.net/>) | To identify the common genes |
| ShinyGO 0.80 (<http://bioinformatics.sdstate.edu/go/>) | Enrichment analysis and Gene ontology (GO) of the screened genes |
| STRING (<https://string-db.org/>) | PPI network analysis |
| Cytoscape v3.10.2 | Visualization of the PPI network |
| SwissADME (<http://www.swissadme.ch/index.php>)  Molsoft (<https://molsoft.com/mprop/>)  ADMETlab 2.0 (<https://admetmesh.scbdd.com/>) | ADMET analysis of the GM |
| CASTp 3.0 (<http://sts.bioe.uic.edu/castp/index.html?2was>) | Active site prediction |
| AutoDockTools- 1.5.7 | Protein-ligand docking |
| Discovery Studio and PyMOL | Visualization of the 2D and 3D protein-ligand interaction. |
| Gromacs2023.1 | Molecular dynamic simulations |

**Table S2** Top 3 gene ontology categories based on fold enrichment scores for cellular components, biological processes, and molecular functions

| **Process** | **GO** | **Classification** | **Genes involved** |
| --- | --- | --- | --- |
| Cellular components | [GO:0016580](http://www.ebi.ac.uk/QuickGO/GTerm?id=GO:0016580#term=history) | Sin3 complex | HDAC1 HDAC2 |
|  | [GO:0016581](http://www.ebi.ac.uk/QuickGO/GTerm?id=GO:0016581#term=history) | NuRD complex | HDAC1 HDAC2 |
|  | [GO:0070822](http://www.ebi.ac.uk/QuickGO/GTerm?id=GO:0070822#term=history) | Sin3-type complex | HDAC1 HDAC2 |
| Biological process | [GO:0032496](http://www.ebi.ac.uk/QuickGO/GTerm?id=GO:0032496#term=history) | Response to lipopolysaccharide | PTGS2 MAPK1 MAPK8 HDAC5 NFKB1 MAPK14 HDAC1 IL1B IL6 TLR4 CYP1A1 CYP1A2 AKT1 HPGD CASP3 CXCL8 RELA JUN HDAC2 |
|  | [GO:0002237](http://www.ebi.ac.uk/QuickGO/GTerm?id=GO:0002237#term=history) | Response to molecule of bacterial origin | PTGS2 MAPK1 MAPK8 HDAC5 NFKB1 MAPK14 HDAC1 IL1B IL6 TLR4 CYP1A1 CYP1A2 AKT1 HPGD CASP3 CXCL8 RELA JUN HDAC2 TLR9 |
|  | [GO:0062197](http://www.ebi.ac.uk/QuickGO/GTerm?id=GO:0062197#term=history) | Cellular response to chemical stress | NOX1 PTGS2 MAPK1 HMOX1 MAPK8 NFE2L2 HDAC1 PPARG IL6 TLR4 AKT1 EGFR G6PD CASP3 RELA JUN NQO1 HDAC2 |
| Molecular function | [GO:0038181](http://www.ebi.ac.uk/QuickGO/GTerm?id=GO:0038181#term=history) | Bile acid receptor activity | NR1H4 VDR GPBAR1 |
|  | [GO:0031078](http://www.ebi.ac.uk/QuickGO/GTerm?id=GO:0031078#term=history) | Histone deacetylase activity (H3-K14 specific) | HDAC9 HDAC4 HDAC5 HDAC1 HDAC8 HDAC11 HDAC3 HDAC2 |
|  | [GO:0101021](http://www.ebi.ac.uk/QuickGO/GTerm?id=GO:0101021#term=history) | Estrogen 2-hydroxylase activity | CYP1A1 CYP1A2 CYP3A4 |

**Table S3** Top 10 significantly enriched pathways identified in the enrichment analysis of RCC

| **Pathway** | **Genes** | **Pathway Genes** | **Fold Enrichment** |
| --- | --- | --- | --- |
| IL-17 signaling pathway | PTGS2, GSK3B, MAPK1, MAPK8, NFKB1, MAPK14, IL1B, IL6, CASP3, CXCL8, RELA, JUN | 12 | 54.47072879 |
| AGE-RAGE signaling pathway in diabetic complications | NOX1, MAPK1, MAPK8, NFKB1, MAPK14, IL1B, IL6, AKT1, CASP3, CXCL8, RELA, JUN | 12 | 50.65777778 |
| Toll-like receptor signaling pathway | MAPK1, MAPK8, NFKB1, MAPK14, IL1B, IL6, TLR4, AKT1, CXCL8, RELA, JUN, TLR9 | 12 | 49.18230852 |
| C-type lectin receptor signaling pathway | PTGS2, MAPK1, MAPK8, NFKB1, IL2, MAPK14, IL1B, IL6, AKT1, PAK1, RELA, JUN | 12 | 48.70940171 |
| TNF signaling pathway | PTGS2, MAPK1, MAPK8, NFKB1, MAPK14, IL1B, IL6, AKT1, CASP3, RELA, JUN | 11 | 41.46097884 |
| T cell receptor signaling pathway | GSK3B, MAPK1, MAPK8, NFKB1, IL2, MAPK14, AKT1, PAK1, RELA, JUN | 10 | 40.9852571 |
| Th17 cell differentiation | RORA, MAPK1, MAPK8, NFKB1, IL2, MAPK14, IL1B, IL6, RELA, JUN | 10 | 39.0877915 |
| Neutrophil extracellular trap formation | HDAC9, MAPK1, HDAC5, NFKB1, MAPK14, HDAC1, TLR4, AKT1, HDAC8, HDAC11, HDAC3, RELA, HDAC2 | 14 | 28.88382066 |
| NOD-like receptor signaling pathway | MAPK1, MAPK8, NFKB1, MAPK14, IL1B, IL6, TLR4, CXCL8, RELA, JUN | 10 | 23.4526749 |
| CAMP signaling pathway | MAPK1, MAPK8, NFKB1, SSTR1, AKT1, PAK1, ADRB2, RELA, JUN, HCAR2, HCAR3 | 11 | 21.20378826 |

**Table S4** Comparative degree values across the M-T-P-D network

| **S.No** | **Pathway** | **Degree score** |
| --- | --- | --- |
| 1 | IL-17 signaling pathway | 13 |
| 2 | Neutrophil extracellular trap formation | 13 |
| 3 | C-type lectin receptor signaling pathway | 13 |
| 4 | Toll-like receptor signaling pathway | 13 |
| 5 | AGE-RAGE signaling pathway in diabetic complications | 13 |
| 6 | CAMP signaling pathway | 12 |
| 7 | TNF signaling pathway | 12 |
| 8 | NOD-like receptor signaling pathway | 11 |
| 9 | Th17 cell differentiation | 11 |
| 10 | T cell receptor signaling pathway | 11 |
| **S.No** | **Genes** | **Degree score** |
| 1 | JUN | 13 |
| 2 | AKTI | 13 |
| 3 | RELA | 12 |
| 4 | MAPK1 | 10 |
| 5 | NFKB1 | 10 |
| 6 | MAPK14 | 9 |
| 7 | MAPK8 | 9 |
| 8 | IL1B | 7 |
| 9 | IL6 | 7 |
| 10 | CXCL8 | 6 |
| 11 | CASP3 | 3 |
| 12 | PAK1 | 3 |
| 13 | IL2 | 3 |
| 14 | PTGS2 | 3 |
| 15 | TLR4 | 3 |
| 16 | GSK3B | 2 |
| 17 | HDAC2 | 1 |
| 18 | TLR9 | 1 |
| 19 | HCAR3 | 1 |
| 20 | HDAC3 | 1 |
| 21 | HDAC8 | 1 |
| 22 | HCAR2 | 1 |
| 23 | HDAC11 | 1 |
| 24 | ADRB2 | 1 |
| 25 | SSTR1 | 1 |
| 26 | HDAC5 | 1 |
| 27 | HDAC9 | 1 |
| 28 | RORA | 1 |
| 29 | NOX1 | 1 |
| 30 | HDAC1 | 1 |
| **S.No** | **Compounds** | **Degree score** |
| 1 | Icaritin | 2 |
| 2 | 3-(3,4-Dihydroxyphenyl)-2-hydroxypropanoic acid | 2 |
| 3 | 4-Hydroxy-(3',4'-dihydroxyphenyl)-valeric acid | 2 |
| 4 | Dihydrogenistein | 2 |
| 5 | Kaempferol | 2 |
| 6 | Apigenin | 2 |
| 7 | Luteolin | 2 |
| 8 | Quercetin | 2 |
| 9 | Nicotinic acid | 1 |
| 10 | 4-Pyridoxic acid | 1 |
| 11 | Pioglitazone | 1 |
| 12 | 5-(3,4-Dihydroxyphenyl)-valerolactone | 1 |
| 13 | Dihydrodaidzein | 1 |
| 14 | Genistein | 1 |
| 15 | Ponciretin | 1 |
| 16 | Naringenin | 1 |
| 17 | 6,7,4'-Trihydroxyisoflavone | 1 |
| 18 | Bile acid | 1 |
| 19 | Deoxycholic acid | 1 |

**Table S5** PPI network analysis of the top 55 genes, highlighting key interactions and their potential implications in the study

| **Confidence** | 0.9 |
| --- | --- |
| **Number of nodes** | 37 |
| **Number of edges** | 90 |
| **Average no. of neighbors** | 4.865 |
| **Avg. local clustering coefficient** | 0.493 |
| **Network density** | 0.135 |
| **Network radius** | 3 |

**Table S6** Top 10 genes identified in the shortest path interaction analysis, ranked according to MNC (Maximum Neighborhood Component) score

| **Rank** | **Name** | **MNC Score** |
| --- | --- | --- |
| 1 | JUN | 13 |
| 2 | RELA | 12 |
| 3 | IL6 | 11 |
| 3 | NFKB1 | 11 |
| 5 | IL1B | 10 |
| 6 | CXCL8 | 8 |
| 7 | AKT1 | 7 |
| 8 | TLR4 | 6 |
| 8 | NFKBIA | 6 |
| 8 | HDAC1 | 6 |

**Table S7** Top 10 genes identified in the shortest path interaction analysis, ranked according to Degree score

| **Rank** | **Name** | **Degree Score** |
| --- | --- | --- |
| 1 | JUN | 26 |
| 2 | IL6 | 24 |
| 2 | RELA | 24 |
| 4 | NFKB1 | 22 |
| 5 | IL1B | 20 |
| 6 | CXCL8 | 18 |
| 7 | AKT1 | 16 |
| 8 | TLR4 | 14 |
| 8 | HDAC1 | 14 |
| 8 | MAPK8 | 14 |

**Table S8** Top 10 genes identified in the shortest path interaction analysis, ranked according to Closeness Score

| **Rank** | **Name** | **Closeness Score** |
| --- | --- | --- |
| 1 | JUN | 23.83333 |
| 2 | RELA | 22.5 |
| 3 | IL6 | 22.16667 |
| 4 | NFKB1 | 22 |
| 5 | IL1B | 20.83333 |
| 6 | CXCL8 | 20.33333 |
| 7 | MAPK8 | 19.33333 |
| 8 | AKT1 | 19.16667 |
| 9 | HDAC1 | 18.91667 |
| 10 | MAPK1 | 18.5 |

**Table S9** Screening of gut microbial metabolites based on Lipinski’s Rule of 5 for drug-likeness assessment

| **Metabolite Name** | **PubChem ID** | **Molecular Weight** | **Canonical smiles** | **Bioavailability score** | **Drug Likeliness** | **TPSA** | **Lipinski's rule** |
| --- | --- | --- | --- | --- | --- | --- | --- |
| 10-[(Acetyloxy)methyl]-9-anthryl)methyl acetate | 613145 | 322.4 g/mol | CC(=O)OCC1=C2C=CC=CC2=C(C3=CC=CC=C31)COC(=O)C | 0.55 | -1 | 52.6 | 0 |
| (20S)-Protopanaxadiol | 11213350 | 460.7 g/mol | CC(=CCCC(C)(C1CCC2(C1C(CC3C2(CCC4C3(CCC(C4(C)C)O)C)C)O)C)O)C | 0.55 | 0.68 | 60.69 | 1 |
| (4S)-4-[(2E)-Oct-2-enoyloxy]-4-(trimethylazaniumyl)butanoate | 53481667 | 285.38 g/mol | CCCCCC=CC(=O)OC(CCC(=O)[O-])[N+](C)(C)C | 0.55 | -0.02 | 66.43 | 0 |
| (R)-3-(4-Hydroxyphenyl)lactate | 9548632 | 181.16 g/mol | C1=CC(=CC=C1CC(C(=O)[O-])O)O | 0.56 | -0.39 | 80.59 | 0 |
| (R)-3-Hydroxybutyrate | 6971058 | 103.10 g/mol | CC(CC(=O)[O-])O | 0.85 | -1.51 | 60.36 | 0 |
| (S)-3-Hydroxybutyric acid | 94318 | 104.10 g/mol | CC(CC(=O)O)O | 0.85 | -1.05 | 57.53 | 0 |
| 1,3-Diphenylpropan-2-ol | 138478 | 212.29 g/mol | C1=CC=C(C=C1)CC(CC2=CC=CC=C2)O | 0.55 | -0.9 | 20.23 | 0 |
| 1-(3,4-Dihydroxyphenyl)-3-(2,4,6-trihydroxyphenyl)-2-propanol | 21722175 | 292.28 g/mol | C1=CC(=C(C=C1CC(CC2=C(C=C(C=C2O)O)O)O)O)O | 0.55 | 0.55 | 121.38 | 1 |
| 1-(3-Chloro-2,6-dihydroxy-4-methoxyphenyl)-1-hexanone | 3081033 | 272.72 g/mol | CCCCCC(=O)C1=C(C(=C(C=C1O)OC)Cl)O | 0.55 | -0.22 | 66.76 | 0 |
| 1-Piperazinepentanamide, N-((1S,2R)-2,3-dihydro-2-hydroxy-1H-inden-1-yl)-2-(((1,1-dimethylethyl)amino)carbonyl)-4-(furo(2,3-b)pyridin-5-ylmethyl)-gamma-hydroxy-alpha-(phenylmethyl)-, (alphaR,gammaS,2S)- | 5481990 | 653.8 g/mol | CC(C)(C)NC(=O)C1CN(CCN1CC(CC(CC2=CC=CC=C2)C(=O)NC3C(CC4=CC=CC=C34)O)O)CC5=CN=C6C(=C5)C=CO6 | 0.55 | 1.55 | 131.17 | 1 |
| 10-Keto-12Z-octadecenoic acid | 24970825 | 296.4 g/mol | CCCCCC=CCC(=O)CCCCCCCCC(=O)O | 0.85 | -0.21 | 54.37 | 0 |
| 10-Oxo-11-octadecenoic acid | 10308378 | 296.4 g/mol | CCCCCCC=CC(=O)CCCCCCCCC(=O)O | 0.85 | -0.42 | 54.37 | 0 |
| 11-Methoxycurvularin | 10381440 | 322.4 g/mol | CC1CCCC(CC(=O)C2=C(CC(=O)O1)C=C(C=C2O)O)OC | 0.55 | 0.32 | 93.06 | 0 |
| 2,2',4,4',5-Pentabromodiphenyl ether | 36159 | 564.7 g/mol | C1=CC(=C(C=C1Br)Br)OC2=CC(=C(C=C2Br)Br)Br | 0.17 | -1.28 | 9.23 | 2 |
| 2,2',4,4'-Tetrabromodiphenyl ether | 95170 | 485.79 g/mol | C1=CC(=C(C=C1Br)Br)OC2=C(C=C(C=C2)Br)Br | 0.55 | -1.34 | 9.23 | 1 |
| 2,3-Bis(3,4-dihydroxybenzyl)butyrolactone | 500189 | 330.3 g/mol | C1C(C(C(=O)O1)CC2=CC(=C(C=C2)O)O)CC3=CC(=C(C=C3)O)O | 0.55 | 0.18 | 107.22 | 0 |
| 2,3-Butanediol | 262 | 90.12 g/mol | CC(C(C)O)O | 0.55 | -1.09 | 40.46 | 0 |
| 2,3-Butanedione | 650 | 86.09 g/mol | CC(=O)C(=O)C | 0.55 | -1.74 | 34.14 | 0 |
| 2,3-Dihydroxypropyl (E)-3-(3,4-dihydroxyphenyl)prop-2-enoate | 5315606 | 254.24 g/mol | C1=CC(=C(C=C1C=CC(=O)OCC(CO)O)O)O | 0.55 | 0.1 | 107.22 | 0 |
| 2,4-Dioxopentanedioic acid | 444873 | 160.08 g/mol | C(C(=O)C(=O)O)C(=O)C(=O)O | 0.56 | -1.38 | 108.74 | 0 |
| 2-(4-Hydroxyphenyl)propionic acid, (2S)- | 6971268 | 166.17 g/mol | CC(C1=CC=C(C=C1)O)C(=O)O | 0.85 | 0.12 | 57.53 | 0 |
| 2-Acetoxypropanoic acid | 79041 | 132.11 g/mol | CC(C(=O)O)OC(=O)C | 0.85 | -1.07 | 63.6 | 0 |
| 2-Amino-1-methyl-6-phenylimidazo[4,5-b]pyridine | 1530 | 224.26 g/mol | CN1C2=C(N=CC(=C2)C3=CC=CC=C3)N=C1N | 0.55 | -0.29 | 56.73 | 0 |
| 2-Hydroxy-3-(4-hydroxyphenyl)propanoic acid | 9378 | 182.17 g/mol | C1=CC(=CC=C1CC(C(=O)O)O)O | 0.56 | -0.14 | 77.76 | 0 |
| 2-Hydroxy-3-(5-hydroxy-1H-indol-3-yl)propanoic acid | 192215 | 221.21 g/mol | C1=CC2=C(C=C1O)C(=CN2)CC(C(=O)O)O | 0.56 | -0.15 | 93.55 | 0 |
| 2-Imino-1-methylimidazolidin-4-one | 588 | 113.12 g/mol | CN1CC(=O)N=C1N | 0.55 | -0.97 | 58.69 | 0 |
| 20(R)-Ginsenoside Rh2 | 54580480 | 622.9 g/mol | CC(=CCCC(C)(C1CCC2(C1C(CC3C2(CCC4C3(CCC(C4(C)C)OC5C(C(C(C(O5)CO)O)O)O)C)C)O)C)O)C | 0.17 | 0.47 | 139.84 | 2 |
| 3,4-Dihydroxy-trans-stilbene | 10176710 | 212.24 g/mol | C1=CC=C(C=C1)C=CC2=CC(=C(C=C2)O)O | 0.55 | -0.7 | 40.46 | 0 |
| 3,4-Dihydroxybenzoic acid | 72 | 154.12 g/mol | C1=CC(=C(C=C1C(=O)O)O)O | 0.56 | 0.23 | 77.76 | 0 |
| 3,4-Dihydroxyphenylacetic acid | 547 | 168.15 g/mol | C1=CC(=C(C=C1CC(=O)O)O)O | 0.56 | -0.04 | 77.76 | 0 |
| 3,9-Dihydroxybenzo[c]chromen-6-one | 10376459 | 228.20 g/mol | C1=CC2=C(C=C1O)C3=C(C=C(C=C3)O)OC2=O | 0.55 | -0.9 | 70.67 | 0 |
| 3-(2,4-Dihydroxyphenyl)propanoic acid | 96384 | 182.17 g/mol | C1=CC(=C(C=C1O)O)CCC(=O)O | 0.56 | -0.2 | 77.76 | 0 |
| 3-(3,4-Dihydroxyphenyl)-2-hydroxypropanoic acid | 439435 | 198.17 g/mol | C1=CC(=C(C=C1CC(C(=O)O)O)O)O | 0.56 | 0.57 | 97.99 | 0 |
| 3-(3-Hydroxyphenyl)propanoic acid | 91 | 166.17 g/mol | C1=CC(=CC(=C1)O)CCC(=O)O | 0.85 | -0.61 | 57.53 | 0 |
| 3-(4-Hydroxyphenyl)propionic acid | 10394 | 166.17 g/mol | C1=CC(=CC=C1CCC(=O)O)O | 0.85 | -0.95 | 57.53 | 0 |
| 3-Hydroxy-4-methoxybenzenepropanoic acid | 2752054 | 196.20 g/mol | COC1=C(C=C(C=C1)CCC(=O)O)O | 0.85 | -0.59 | 66.76 | 0 |
| 3-Hydroxybenzoic acid | 7420 | 138.12 g/mol | C1=CC(=CC(=C1)O)C(=O)O | 0.85 | -0.6 | 57.53 | 0 |
| 3-Hydroxyphenethyl alcohol | 83404 | 138.16 g/mol | C1=CC(=CC(=C1)O)CCO | 0.55 | -0.81 | 40.46 | 0 |
| 3-Indolepropionic acid | 3744 | 189.21 g/mol | C1=CC=C2C(=C1)C(=CN2)CCC(=O)O | 0.85 | -1.37 | 53.09 | 0 |
| 3-Methylindole | 6736 | 131.17 g/mol | CC1=CNC2=CC=CC=C12 | 0.55 | -1.85 | 15.79 | 0 |
| 3-Methyloxindole | 150923 | 147.17 g/mol | CC1C2=CC=CC=C2NC1=O | 0.55 | -0.71 | 29.1 | 0 |
| 3-Phenylpropionic acid | 107 | 150.17 g/mol | C1=CC=C(C=C1)CCC(=O)O | 0.85 | -1.23 | 37.3 | 0 |
| 4-Hydroxy-(3',4'-dihydroxyphenyl)-valeric acid | 52920332 | 226.23 g/mol | C1=CC(=C(C=C1CC(CCC(=O)O)O)O)O | 0.56 | 0.66 | 97.99 | 0 |
| 4-Hydroxybenzoic acid | 135 | 138.12 g/mol | C1=CC(=CC=C1C(=O)O)O | 0.85 | -0.37 | 57.53 | 0 |
| 4-Hydroxyphenylacetic acid | 127 | 152.15 g/mol | C1=CC(=CC=C1CC(=O)O)O | 0.85 | -0.83 | 57.53 | 0 |
| 4-Pyridoxic acid | 6723 | 183.16 g/mol | CC1=NC=C(C(=C1O)C(=O)O)CO | 0.56 | 0.44 | 90.65 | 0 |
| 5-(3,4-Dihydroxyphenyl)-valerolactone | 45093073 | 208.21 g/mol | C1CC(OC(=O)C1)C2=CC(=C(C=C2)O)O | 0.55 | 0.61 | 66.76 | 0 |
| 5-Hydroxy-1H-imidazole-4-carboxamide | 124343 | 127.10 g/mol | C1=NC(=C(N1)C(=O)N)O | 0.55 | -0.28 | 92 | 0 |
| 5-Hydroxyindole-3-acetic acid | 1826 | 191.18 g/mol | C1=CC2=C(C=C1O)C(=CN2)CC(=O)O | 0.85 | -0.81 | 73.32 | 0 |
| 5-OH-Equol | 9795113 | 258.27 g/mol | C1C(COC2=CC(=CC(=C21)O)O)C3=CC=C(C=C3)O | 0.55 | 0.17 | 69.92 | 0 |
| 6'-Hydroxy-O-desmethylangolensin | 20601635 | 274.27 g/mol | CC(C1=CC=C(C=C1)O)C(=O)C2=C(C=C(C=C2O)O)O | 0.55 | 1 | 97.99 | 0 |
| 6,7,4'-Trihydroxyisoflavone | 5284649 | 270.24 g/mol | C1=CC(=CC=C1C2=COC3=CC(=C(C=C3C2=O)O)O)O | 0.55 | 0.4 | 90.9 | 0 |
| 7-Hydroxy-5-methyl-3-phenyl-6,7,8,9-tetrahydropyrido[3',2':4,5]imidazo[1,2-a]pyrimidin-5-ium chloride | 129849310 | 316.78 g/mol | CN1C2=C(N=CC(=C2)C3=CC=CC=C3)[N+]4=C1NC(CC4)O.[Cl-] | 0.55 | 0.23 | 53.96 | 0 |
| 8-Prenylnaringenin | 480764 | 340.4 g/mol | CC(=CCC1=C2C(=C(C=C1O)O)C(=O)CC(O2)C3=CC=C(C=C3)O)C | 0.55 | 1.36 | 86.99 | 0 |
| Acacetin | 5280442 | 284.26 g/mol | COC1=CC=C(C=C1)C2=CC(=O)C3=C(C=C(C=C3O2)O)O | 0.55 | 0.2 | 79.9 | 0 |
| Acetate | 175 | 59.04 g/mol | CC(=O)[O-] | 0.85 | -1.07 | 40.13 | 0 |
| Acetoin | 179 | 88.11 g/mol | CC(C(=O)C)O | 0.55 | -1 | 37.3 | 0 |
| Acetyl phosphate(2-) | 4183249 | 138.02 g/mol | CC(=O)OP(=O)([O-])[O-] | 0.56 | -1.34 | 99.3 | 0 |
| Acifran | 51576 | 218.20 g/mol | CC1(C(=O)C=C(O1)C(=O)O)C2=CC=CC=C2 | 0.85 | -0.23 | 63.6 | 0 |
| Alanine | 5950 | 89.09 g/mol | CC(C(=O)O)N | 0.55 | -0.96 | 63.32 | 0 |
| Apigenin | 5280443 | 270.24 g/mol | C1=CC(=CC=C1C2=CC(=O)C3=C(C=C(C=C3O2)O)O)O | 0.55 | 0.39 | 90.9 | 0 |
| Arctigenin | 64981 | 372.4 g/mol | COC1=C(C=C(C=C1)CC2COC(=O)C2CC3=CC(=C(C=C3)O)OC)OC | 0.55 | 0.04 | 74.22 | 0 |
| Asparagine | 6267 | 132.12 g/mol | C(C(C(=O)O)N)C(=O)N | 0.55 | -0.05 | 106.41 | 0 |
| Baicalein | 5281605 | 270.24 g/mol | C1=CC=C(C=C1)C2=CC(=O)C3=C(O2)C=C(C(=C3O)O)O | 0.55 | -0.1 | 90.9 | 0 |
| Baicalin | 64982 | 446.4 g/mol | C1=CC=C(C=C1)C2=CC(=O)C3=C(C(=C(C=C3O2)OC4C(C(C(C(O4)C(=O)O)O)O)O)O)O | 0.11 | 0.58 | 187.12 | 2 |
| Baohuoside I | 5488822 | 514.5 g/mol | CC1C(C(C(C(O1)OC2=C(OC3=C(C(=CC(=C3C2=O)O)O)CC=C(C)C)C4=CC=C(C=C4)OC)O)O)O | 0.55 | 0.9 | 159.05 | 1 |
| beta-D-Fructofuranose | 439709 | 180.16 g/mol | C(C1C(C(C(O1)(CO)O)O)O)O | 0.55 | -0.97 | 110.38 | 0 |
| beta-D-Gal-(1->4)-beta-D-GlcNAc-(1->3)-beta-D-Gal-(1->4)-D-Glc | 9831622 | 707.6 g/mol | CC(=O)NC1C(C(C(OC1OC2C(C(OC(C2O)OC3C(OC(C(C3O)O)O)CO)CO)O)CO)OC4C(C(C(C(O4)CO)O)O)O)O | 0.17 | 0.27 | 356.7 | 3 |
| Bile acid | 439520 | 408.6 g/mol | CC(CCC(=O)O)C1CCC2C1(C(CC3C2C(CC4C3(CCC(C4)O)C)O)O)C | 0.56 | 0.43 | 97.99 | 0 |
| Butyrate | 104775 | 87.10 g/mol | CCCC(=O)[O-] | 0.85 | -1.61 | 40.13 | 0 |
| Caffeic acid | 689043 | 180.16 g/mol | C1=CC(=C(C=C1C=CC(=O)O)O)O | 0.56 | -0.35 | 77.76 | 0 |
| Chrysin | 5281607 | 254.24 g/mol | C1=CC=C(C=C1)C2=CC(=O)C3=C(C=C(C=C3O2)O)O | 0.55 | -0.21 | 70.67 | 0 |
| Citric acid | 311 | 192.12 g/mol | C(C(=O)O)C(CC(=O)O)(C(=O)O)O | 0.56 | 0.52 | 132.13 | 0 |
| Colibactin | 138805674 | 770.9 g/mol | CC1CCC(=N1)C2=C(C3(CC3)NC2=O)CC(=O)NCC4=NC(=CS4)C(=O)C(=O)C5=NC(=CS5)C(=O)CNC(=O)CC6=C(C(=O)NC67CC7)C8=NC(CC8)C | 0.17 | 0.59 | 274.59 | 2 |
| Creatine | 586 | 131.13 g/mol | CN(CC(=O)O)C(=N)N | 0.55 | -0.72 | 90.41 | 0 |
| Cystine | 595 | 240.3 g/mol | C(C(C(=O)O)N)SSCC(C(=O)O)N | 0.55 | -0.83 | 177.24 | 0 |
| D-Glucuronic Acid | 94715 | 194.14 g/mol | C1(C(C(OC(C1O)O)C(=O)O)O)O | 0.56 | -0.3 | 127.45 | 0 |
| D-Lactic acid | 61503 | 90.08 g/mol | CC(C(=O)O)O | 0.85 | -0.2 | 57.53 | 0 |
| D-Mannose | 18950 | 180.16 g/mol | C(C1C(C(C(C(O1)O)O)O)O)O | 0.55 | -0.12 | 110.38 | 0 |
| Daidzein | 5281708 | 254.24 g/mol | C1=CC(=CC=C1C2=COC3=C(C2=O)C=CC(=C3)O)O | 0.55 | 0.29 | 70.67 | 0 |
| Deoxycholic acid | 222528 | 392.6 g/mol | CC(CCC(=O)O)C1CCC2C1(C(CC3C2CCC4C3(CCC(C4)O)C)O)C | 0.56 | 0.31 | 77.76 | 0 |
| Dihydrocaffeic acid | 348154 | 182.17 g/mol | C1=CC(=C(C=C1CCC(=O)O)O)O | 0.56 | 0.08 | 77.76 | 0 |
| Dihydrodaidzein | 176907 | 256.25 g/mol | C1C(C(=O)C2=C(O1)C=C(C=C2)O)C3=CC=C(C=C3)O | 0.55 | 0.82 | 66.76 | 0 |
| Dihydrogenistein | 9838356 | 272.25 g/mol | C1C(C(=O)C2=C(C=C(C=C2O1)O)O)C3=CC=C(C=C3)O | 0.55 | 0.92 | 86.99 | 0 |
| Dihydroglycitein | 101101166 | 286.28 g/mol | COC1=C(C=C2C(=C1)C(=O)C(CO2)C3=CC=C(C=C3)O)O | 0.55 | 0.79 | 75.99 | 0 |
| Dihydroresveratrol | 185914 | 230.26 g/mol | C1=CC(=CC=C1CCC2=CC(=CC(=C2)O)O)O | 0.55 | -0.49 | 60.69 | 0 |
| Diosgenin | 99474 | 414.6 g/mol | CC1CCC2(C(C3C(O2)CC4C3(CCC5C4CC=C6C5(CCC(C6)O)C)C)C)OC1 | 0.55 | -0.09 | 38.69 | 1 |
| Diosmetin | 5281612 | 300.26 g/mol | COC1=C(C=C(C=C1)C2=CC(=O)C3=C(C=C(C=C3O2)O)O)O | 0.55 | 0.06 | 100.13 | 0 |
| DL-Alanine | 602 | 89.09 g/mol | CC(C(=O)O)N | 0.55 | -0.96 | 63.32 | 0 |
| Dopamine | 681 | 153.18 g/mol | C1=CC(=C(C=C1CCN)O)O | 0.55 | 0.09 | 66.48 | 0 |
| Enterodiol | 115089 | 302.4 g/mol | C1=CC(=CC(=C1)O)CC(CO)C(CC2=CC(=CC=C2)O)CO | 0.55 | -0.02 | 80.92 | 0 |
| Equol | 91469 | 242.27 g/mol | C1C(COC2=C1C=CC(=C2)O)C3=CC=C(C=C3)O | 0.55 | -0.07 | 49.69 | 0 |
| Ethanol | 702 | 46.07 g/mol | CCO | 0.55 | -0.62 | 20.23 | 0 |
| Ethyl phenyllactate, (-)- | 9877619 | 194.23 g/mol | CCOC(=O)C(CC1=CC=CC=C1)O | 0.55 | -0.74 | 46.53 | 0 |
| Folic acid | 135398658 | 441.4 g/mol | C1=CC(=CC=C1C(=O)NC(CCC(=O)O)C(=O)O)NCC2=CN=C3C(=N2)C(=O)NC(=N3)N | 0.11 | 1.09 | 213.28 | 2 |
| Formate | 283 | 45.017 g/mol | C(=O)[O-] | 0.85 | -1.02 | 40.13 | 0 |
| Genipin | 442424 | 226.23 g/mol | COC(=O)C1=COC(C2C1CC=C2CO)O | 0.56 | -0.44 | 75.99 | 0 |
| Genistein | 5280961 | 270.24 g/mol | C1=CC(=CC=C1C2=COC3=CC(=CC(=C3C2=O)O)O)O | 0.55 | 0.44 | 90.9 | 0 |
| Ginsenoside Rh2 | 119307 | 622.9 g/mol | CC(=CCCC(C)(C1CCC2(C1C(CC3C2(CCC4C3(CCC(C4(C)C)OC5C(C(C(C(O5)CO)O)O)O)C)C)O)C)O)C | 0.17 | 0.47 | 139.84 | 2 |
| Ginsenoside-Rd | 24721561 | 963.2 g/mol | CC(=CCCC(C)(C1CCC2(C1C(CC3C2(CC(C4C3(CCC(C4(C)C)O)C)OC5C(C(C(C(O5)CO)O)O)OC6C(C(C(C(O6)CO)O)O)O)C)O)C)OC7C(C(C(C(O7)CO)O)O)O)C | 0.17 | 0.36 | 318.37 | 3 |
| Glutamic acid | 33032 | 147.13 g/mol | C(CC(=O)O)C(C(=O)O)N | 0.56 | 0.24 | 100.62 | 0 |
| Glutathione | 124886 | 307.33 g/mol | C(CC(=O)NC(CS)C(=O)NCC(=O)O)C(C(=O)O)N | 0.11 | -0.35 | 197.62 | 0 |
| Glycerol | 753 | 92.09 g/mol | C(C(CO)O)O | 0.55 | -0.99 | 60.69 | 0 |
| Glycine | 750 | 75.07 g/mol | C(C(=O)O)N | 0.55 | -0.16 | 63.32 | 0 |
| Glycitein | 5317750 | 284.26 g/mol | COC1=C(C=C2C(=C1)C(=O)C(=CO2)C3=CC=C(C=C3)O)O | 0.55 | 0.29 | 79.9 | 0 |
| Glycocholic acid | 10140 | 465.6 g/mol | CC(CCC(=O)NCC(=O)O)C1CCC2C1(C(CC3C2C(CC4C3(CCC(C4)O)C)O)O)C | 0.56 | 0.29 | 127.09 | 0 |
| Hesperetin dihydrochalcone | 147608 | 304.29 g/mol | COC1=C(C=C(C=C1)CCC(=O)C2=C(C=C(C=C2O)O)O)O | 0.55 | -0.06 | 107.22 | 0 |
| Histidine | 6274 | 155.15 g/mol | C1=C(NC=N1)CC(C(=O)O)N | 0.55 | -0.21 | 92 | 0 |
| Hydrogen | 783 | 2.016 g/mol | [HH] | 0.55 | -1.56 | 0 | 0 |
| Hydroquinone | 785 | 110.11 g/mol | C1=CC(=CC=C1O)O | 0.55 | -1.16 | 40.46 | 0 |
| Hydroxyquercitrin | 129887767 | 464.4 g/mol | CC1C(C(C(C(O1)OC2=C(OC3=C(C2=O)C(=C(C(=C3)O)O)O)C4=CC(=C(C=C4)O)O)O)O)O | 0.17 | 0.74 | 210.51 | 2 |
| Icaritin | 5318980 | 368.4 g/mol | CC(=CCC1=C2C(=C(C=C1O)O)C(=O)C(=C(O2)C3=CC=C(C=C3)OC)O)C | 0.55 | 0.84 | 100.13 | 0 |
| Indole | 798 | 117.15 g/mol | C1=CC=C2C(=C1)C=CN2 | 0.55 | -2.75 | 15.79 | 0 |
| Indole-3-acrylic acid | 5375048 | 187.19 g/mol | C1=CC=C2C(=C1)C(=CN2)C=CC(=O)O | 0.85 | -1.87 | 53.09 | 0 |
| Indole-3-carboxaldehyde | 10256 | 145.16 g/mol | C1=CC=C2C(=C1)C(=CN2)C=O | 0.55 | -2.33 | 32.86 | 0 |
| Indole-3-carboxylic acid | 69867 | 161.16 g/mol | C1=CC=C2C(=C1)C(=CN2)C(=O)O | 0.85 | -1.35 | 53.09 | 0 |
| Indole-3-lactic acid | 92904 | 205.21 g/mol | C1=CC=C2C(=C1)C(=CN2)CC(C(=O)O)O | 0.85 | -0.84 | 73.32 | 0 |
| Indoxyl sulfate | 10258 | 213.21 g/mol | C1=CC=C2C(=C1)C(=CN2)OS(=O)(=O)O | 0.56 | -1.83 | 87.77 | 0 |
| Isobutyric acid | 6590 | 88.11 g/mol | CC(C)C(=O)O | 0.85 | -1.18 | 37.3 | 0 |
| Isoquercitrin | 5280804 | 464.4 g/mol | C1=CC(=C(C=C1C2=C(C(=O)C3=C(C=C(C=C3O2)O)O)OC4C(C(C(C(O4)CO)O)O)O)O)O | 0.17 | 0.68 | 210.51 | 2 |
| Isovaleric acid | 10430 | 102.13 g/mol | CC(C)CC(=O)O | 0.85 | -1.51 | 37.3 | 0 |
| Kaempferol | 5280863 | 286.24 g/mol | C1=CC(=CC=C1C2=C(C(=O)C3=C(C=C(C=C3O2)O)O)O)O | 0.55 | 0.5 | 111.13 | 0 |
| Kynurenic acid | 3845 | 189.17 g/mol | C1=CC=C2C(=C1)C(=O)C=C(N2)C(=O)O | 0.85 | -0.01 | 70.16 | 0 |
| L-Cysteine | 5862 | 121.16 g/mol | C(C(C(=O)O)N)S | 0.55 | -0.91 | 102.12 | 0 |
| l-Isoleucine | 6306 | 131.17 g/mol | CCC(C)C(C(=O)O)N | 0.55 | -1.02 | 63.32 | 0 |
| L-Lactate | 5460161 | 89.07 g/mol | CC(C(=O)[O-])O | 0.85 | -1.09 | 60.36 | 0 |
| L-Threonine | 6288 | 119.12 g/mol | CC(C(C(=O)O)N)O | 0.55 | -0.63 | 83.55 | 0 |
| Lactate | 91435 | 89.07 g/mol | CC(C(=O)[O-])O | 0.85 | -1.09 | 60.36 | 0 |
| Lacto-N-tetraose | 440993 | 707.6 g/mol | CC(=O)NC1C(C(C(OC1OC2C(C(OC(C2O)OC3C(OC(C(C3O)O)O)CO)CO)O)CO)O)OC4C(C(C(C(O4)CO)O)O)O | 0.17 | 0.27 | 356.7 | 3 |
| Lariciresinol | 332427 | 360.4 g/mol | COC1=C(C=CC(=C1)CC2COC(C2CO)C3=CC(=C(C=C3)O)OC)O | 0.55 | 0.29 | 88.38 | 0 |
| Leucine | 6106 | 131.17 g/mol | CC(C)CC(C(=O)O)N | 0.55 | -0.68 | 63.32 | 0 |
| Leucocianidol | 71629 | 306.27 g/mol | C1=CC(=C(C=C1C2C(C(C3=C(C=C(C=C3O2)O)O)O)O)O)O | 0.55 | 0.53 | 130.61 | 1 |
| Levodopa | 6047 | 197.19 g/mol | C1=CC(=C(C=C1CC(C(=O)O)N)O)O | 0.55 | 0.58 | 103.78 | 0 |
| Lithocholic acid | 9903 | 376.6 g/mol | CC(CCC(=O)O)C1CCC2C1(CCC3C2CCC4C3(CCC(C4)O)C)C | 0.85 | 0.37 | 57.53 | 1 |
| Loganetin | 10466307 | 228.24 g/mol | CC1C(CC2C1C(OC=C2C(=O)OC)O)O | 0.56 | -0.44 | 75.99 | 0 |
| Lunularin | 181511 | 214.26 g/mol | C1=CC(=CC(=C1)O)CCC2=CC=C(C=C2)O | 0.55 | -0.33 | 40.46 | 0 |
| Luteolin | 5280445 | 286.24 g/mol | C1=CC(=C(C=C1C2=CC(=O)C3=C(C=C(C=C3O2)O)O)O)O | 0.55 | 0.38 | 111.13 | 0 |
| Malic acid | 525 | 134.09 g/mol | C(C(C(=O)O)O)C(=O)O | 0.56 | -0.5 | 94.83 | 0 |
| Myricetin | 5281672 | 318.23 g/mol | C1=C(C=C(C(=C1O)O)O)C2=C(C(=O)C3=C(C=C(C=C3O2)O)O)O | 0.55 | -0.24 | 151.59 | 1 |
| Naringenin | 439246 | 272.25 g/mol | C1C(OC2=CC(=CC(=C2C1=O)O)O)C3=CC=C(C=C3)O | 0.55 | 0.82 | 86.99 | 0 |
| Naringenin chalcone | 5280960 | 272.25 g/mol | C1=CC(=CC=C1C=CC(=O)C2=C(C=C(C=C2O)O)O)O | 0.55 | -0.23 | 97.99 | 0 |
| Nicotinic acid | 938 | 123.11 g/mol | C1=CC(=CN=C1)C(=O)O | 0.85 | 0.3 | 50.19 | 0 |
| Norathyriol | 5281656 | 260.20 g/mol | C1=C(C=C2C(=C1O)C(=O)C3=CC(=C(C=C3O2)O)O)O | 0.55 | -0.6 | 111.13 | 0 |
| O-Desmethylangolensin | 89472 | 258.27 g/mol | CC(C1=CC=C(C=C1)O)C(=O)C2=C(C=C(C=C2)O)O | 0.55 | 1.2 | 77.76 | 0 |
| Oxalacetic acid | 970 | 132.07 g/mol | C(C(=O)C(=O)O)C(=O)O | 0.56 | -1.23 | 91.67 | 0 |
| Oxindole | 321710 | 133.15 g/mol | C1C2=CC=CC=C2NC1=O | 0.55 | -1.32 | 29.1 | 0 |
| p-Cresol glucuronide | 154035 | 284.26 g/mol | CC1=CC=C(C=C1)OC2C(C(C(C(O2)C(=O)O)O)O)O | 0.56 | -1.15 | 116.45 | 0 |
| p-Cresol sulfate | 4615423 | 188.20 g/mol | CC1=CC=C(C=C1)OS(=O)(=O)O | 0.85 | -1.52 | 71.98 | 0 |
| Palmitic acid | 985 | 256.42 g/mol | CCCCCCCCCCCCCCCC(=O)O | 0.85 | -0.54 | 37.3 | 1 |
| Phenylacetic acid | 999 | 136.15 g/mol | C1=CC=C(C=C1)CC(=O)O | 0.85 | -1.32 | 37.3 | 0 |
| Phenylacetylglutamine | 92258 | 264.28 g/mol | C1=CC=C(C=C1)CC(=O)NC(CCC(=O)N)C(=O)O | 0.56 | -0.13 | 109.49 | 0 |
| Phenylalanine | 6140 | 165.19 g/mol | C1=CC=C(C=C1)CC(C(=O)O)N | 0.55 | -0.45 | 63.32 | 0 |
| Phloretin | 4788 | 274.27 g/mol | C1=CC(=CC=C1CCC(=O)C2=C(C=C(C=C2O)O)O)O | 0.55 | 0.09 | 97.99 | 0 |
| Phloroglucinol | 359 | 126.11 g/mol | C1=C(C=C(C=C1O)O)O | 0.55 | -1.05 | 60.69 | 0 |
| Pioglitazone | 4829 | 356.4 g/mol | CCC1=CN=C(C=C1)CCOC2=CC=C(C=C2)CC3C(=O)NC(=O)S3 | 0.55 | 0.94 | 93.59 | 0 |
| Pipecolic acid | 849 | 129.16 g/mol | C1CCNC(C1)C(=O)O | 0.55 | -1.26 | 49.33 | 0 |
| Platycodin D | 162859 | 1225.3 g/mol | CC1C(C(C(C(O1)OC2C(C(COC2OC(=O)C34CCC(CC3C5=CCC6C(C5(CC4O)C)(CCC7C6(CC(C(C7(CO)CO)OC8C(C(C(C(O8)CO)O)O)O)O)C)C)(C)C)O)O)O)O)OC9C(C(C(CO9)O)OC1C(C(CO1)(CO)O)O)O | 0.55 | 0.9 | 453.28 | 1 |
| Ponciretin | 25201019 | 285.27 g/mol | COC1=CC=C(C=C1)C2CC(=O)C3=C(C=C(C=C3O2)O)[O-] | 0.56 | 0.58 | 78.82 | 0 |
| Proline | 145742 | 115.13 g/mol | C1CC(NC1)C(=O)O | 0.55 | -1.02 | 49.33 | 0 |
| Propanol | 1031 | 60.10 g/mol | CCCO | 0.55 | -0.67 | 20.23 | 0 |
| Propionate | 104745 | 73.07 g/mol | CCC(=O)[O-] | 0.85 | -1.23 | 40.13 | 0 |
| Protopanaxadiol | 9920281 | 460.7 g/mol | CC(=CCCC(C)(C1CCC2(C1C(CC3C2(CCC4C3(CCC(C4(C)C)O)C)C)O)C)O)C | 0.55 | 0.68 | 60.69 | 1 |
| Pyruvate | 107735 | 87.05 g/mol | CC(=O)C(=O)[O-] | 0.85 | -1.77 | 57.2 | 0 |
| Quercetin | 5280343 | 302.23 g/mol | C1=CC(=C(C=C1C2=C(C(=O)C3=C(C=C(C=C3O2)O)O)O)O)O | 0.55 | 0.52 | 131.36 | 0 |
| Quercimeritrin | 5282160 | 464.4 g/mol | C1=CC(=C(C=C1C2=C(C(=O)C3=C(C=C(C=C3O2)OC4C(C(C(C(O4)CO)O)O)O)O)O)O)O | 0.17 | 0.68 | 210.51 | 2 |
| Quercitrin | 5280459 | 448.4 g/mol | CC1C(C(C(C(O1)OC2=C(OC3=CC(=CC(=C3C2=O)O)O)C4=CC(=C(C=C4)O)O)O)O)O | 0.17 | 0.82 | 190.28 | 2 |
| Quinic acid | 6508 | 192.17 g/mol | C1C(C(C(CC1(C(=O)O)O)O)O)O | 0.56 | -1.06 | 118.22 | 0 |
| Secoisolariciresinol | 65373 | 362.4 g/mol | COC1=C(C=CC(=C1)CC(CO)C(CC2=CC(=C(C=C2)O)OC)CO)O | 0.55 | -0.4 | 99.38 | 0 |
| Sedoheptulose | 5459879 | 210.18 g/mol | C(C(C(C(C(C(=O)CO)O)O)O)O)O | 0.55 | 0.44 | 138.45 | 1 |
| Serine | 5951 | 105.09 g/mol | C(C(C(=O)O)N)O | 0.55 | -1.04 | 83.55 | 0 |
| Serotonin | 5202 | 176.21 g/mol | C1=CC2=C(C=C1O)C(=CN2)CCN | 0.55 | -0.98 | 62.04 | 0 |
| Sodium 3-hydroxybutyrate | 23676771 | 126.09 g/mol | CC(CC(=O)[O-])O.[Na+] | 0.55 | -1.15 | 60.36 | 0 |
| Succinate | 160419 | 116.07 g/mol | C(CC(=O)[O-])C(=O)[O-] | 0.56 | -0.97 | 80.26 | 0 |
| Sulfate | 1117 | 96.07 g/mol | [O-]S(=O)(=O)[O-] | 0.56 | -1.36 | 88.64 | 0 |
| Tartaric acid | 875 | 150.09 g/mol | C(C(C(=O)O)O)(C(=O)O)O | 0.56 | 0.59 | 115.06 | 0 |
| Taurocholic acid | 6675 | 515.7 g/mol | CC(CCC(=O)NCCS(=O)(=O)O)C1CCC2C1(C(CC3C2C(CC4C3(CCC(C4)O)C)O)O)C | 0.11 | -0.27 | 152.54 | 1 |
| Tretinoin | 444795 | 300.4 g/mol | CC1=C(C(CCC1)(C)C)C=CC(=CC=CC(=CC(=O)O)C)C | 0.85 | 0.71 | 37.3 | 1 |
| Tricarballylic acid | 14925 | 176.12 g/mol | C(C(CC(=O)O)C(=O)O)C(=O)O | 0.56 | -0.53 | 111.9 | 0 |
| Trimethylamine | 1146 | 59.11 g/mol | CN(C)C | 0.55 | -1.16 | 3.24 | 0 |
| Trimethylamine oxide | 1145 | 75.11 g/mol | C[N+](C)(C)[O-] | 0.55 | -1.15 | 29.43 | 0 |
| Urolithin A | 5488186 | 228.20 g/mol | C1=CC2=C(C=C1O)C(=O)OC3=C2C=CC(=C3)O | 0.55 | -0.9 | 70.67 | 0 |
| Ursodeoxycholic acid | 31401 | 392.6 g/mol | CC(CCC(=O)O)C1CCC2C1(CCC3C2C(CC4C3(CCC(C4)O)C)O)C | 0.56 | 0.45 | 77.76 | 0 |
| Valerate | 114781 | 101.12 g/mol | CCCCC(=O)[O-] | 0.85 | -1.13 | 40.13 | 0 |
| Vancomycin | 14969 | 1449.2 g/mol | CC1C(C(CC(O1)OC2C(C(C(OC2OC3=C4C=C5C=C3OC6=C(C=C(C=C6)C(C(C(=O)NC(C(=O)NC5C(=O)NC7C8=CC(=C(C=C8)O)C9=C(C=C(C=C9O)O)C(NC(=O)C(C(C1=CC(=C(O4)C=C1)Cl)O)NC7=O)C(=O)O)CC(=O)N)NC(=O)C(CC(C)C)NC)O)Cl)CO)O)O)(C)N)O |  |  |  |  |

**Table S10** Comprehensive molecular docking analysis of JUN protein interactions with the screened gut microbial metabolites

| **Ligand** | **PubChem ID** | **Binding Energy (kcal/mol)** | **H- bond interaction (Amino acid residues)** | **Other interaction (Amino acid residues)** |
| --- | --- | --- | --- | --- |
| Deoxycholic acid | 222528 | -5.7 | THR91, THR93 | ILE88, THR89, THR90, PRO94, THR95, GLN96, PHE97, LEU98 |
| Ursodeoxycholic acid | 31401 | -5.7 | ASN172, ASN175 | VAL169, PHE176, ASN177, PRO178, GLY179, ALA180, LEU181 |
| Bile acid | 439520 | -5.7 | GLN82, PRO94 | ILE88, THR89, THR90, THR91, PRO92, THR93, THR95, GLN96, PHE97, LEU98 |
| 6,7,4'-Trihydroxyisoflavone | 5284649 | -5.7 | ASN177 | VAL169, TYR170, ALA171, ASN172, ASN175, PHE176, GLY179, ALA180, LEU181 |
| 8-Prenylnaringenin | 480764 | -5.7 | - | VAL169, TYR170, ALA171, ASN172, LEU173, ASN175, PHE176, ASN177, LEU181 |
| Naringenin | 439246 | -5.6 | - | VAL169, TYR170, ALA171, ASN172, LEU173, ASN175, PHE176, ASN177 |
| Apigenin | 5280443 | -5.5 | - | VAL169, TYR170, ALA171, ASN172, ASN175, PHE176, ASN177, ALA180 |
| Kaempferol | 5280863 | -5.5 | - | VAL169, TYR170, ALA171, ASN172, LEU173, ASN175, PHE176, ASN177 |
| Dihydrogenistein | 9838356 | -5.4 | SER48, LEU49, LYS50 | ASP44, PRO45, VAL46, GLY47, PRO51, HIS52, LEU53, ARG54 |
| Ponciretin | 25201019 | -5.3 | - | ALA278, GLU281, GLU282, VAL284, LYS285, LYS288 |
| Genistein | 5280961 | -5.1 | VAL66, SER73, GLU77 | GLY67, LEU69, LYS70, LEU71, ALA72, PRO74, GLU75, LEU76 |
| 11-Methoxycurvularin | 10381440 | -5.0 | LEU49, LEU53 | PRO45, VAL46, GLY47, SER48, PRO51, ARG54 |
| Dihydrodaidzein | 176907 | -5.0 | GLY47, SER48 | ASP44, PRO45, VAL46, LEU49, ARG54 |
| Dihydroglycitein | 101101166 | -4.9 | - | ILE88, THR89, THR90, THR91, THR93, PRO94, THR95, GLN96, PHE97, LEU98 |
| 5-(3,4-Dihydroxyphenyl)-valerolactone | 45093073 | -4.7 | ASN85, HIS87 | ARG78, ILE81, GLN82, GLY86, ILE88 |
| Pioglitazone | 4829 | -4.7 | - | GLN248, GLU251, ARG252, LYS254, ALA255, LYS258 |
| 6'-Hydroxy-O-desmethylangolensin | 20601635 | -4.6 | ASN262, ARG263 | ARG259, ALA266, ARG270 |
| O-Desmethylangolensin | 89472 | -4.6 | - | PHE114, VAL115, LEU118, ALA119, HIS122, TYR170 |
| Levodopa | 6047 | -4.2 | THR91, PRO94, PHE97 | ILE88, THR89, THR93, THR95, GLN96, LEU98 |
| 3-(3,4-Dihydroxyphenyl)-2-hydroxypropanoic acid | 439435 | -4.1 | THR89, THR91, PHE97 | ILE88, THR90, THR93, PRO94, THR95, GLN96, LEU98 |
| 4-Hydroxy-(3',4'-dihydroxyphenyl)-valeric acid | 52920332 | -4.1 | THR91, PRO94, PHE97, LEU98 | ILE88, THR89, THR93, THR95, GLN96 |
| 4-Pyridoxic acid | 6723 | -4.1 | THR89, THR91, THR93 | ILE88, THR90, PRO92, PRO94, THR95 |
| Citric acid | 311 | -3.9 | THR91, THR93, THR95 | THR89, THR90, PRO92, PRO94 |
| Tartaric acid | 875 | -3.7 | THR89, THR91, THR93 | THR90, PRO92, THR95 |
| Nicotinic acid | 938 | -3.4 | ASN177 | ASN175, PHE176 |
